# Construction of a Searchable Database for Gene Expression Changes in Spinal Cord Injury Experiments

**DOI:** 10.1089/neu.2023.0035

**Published:** 2024-05-25

**Authors:** Eric C. Rouchka, Carlos de Almeida, Randi B. House, Jonah C. Daneshmand, Julia H. Chariker, Sujata Saraswat-Ohri, Cynthia Gomes, Morgan Sharp, Alice Shum-Siu, Greta M. Cesarz, Jeffrey C. Petruska, David S. K. Magnuson

**Affiliations:** ^1^ Department of Biochemistry and Molecular Genetics, University of Louisville, Louisville, Kentucky, USA.; ^2^ Kentucky IDeA Networks of Biomedical Research Excellence (KY INBRE) Bioinformatics Core, University of Louisville, Louisville, Kentucky, USA.; ^3^ Bioinformatics Program, University of Louisville, Louisville, Kentucky, USA.; ^4^ Translational Neuroscience Program, University of Louisville, Louisville, Kentucky, USA.; ^5^ Kentucky Spinal Cord Injury Research Center, University of Louisville, Louisville, Kentucky, USA.; ^6^ Department of Bioengineering, University of Louisville, Louisville, Kentucky, USA.; ^7^ Department of Neuroscience Training, University of Louisville, Louisville, Kentucky, USA.; ^8^ Department of Neurological Surgery, University of Louisville, Louisville, Kentucky, USA.; ^9^ Department of Anatomical Sciences and Neurobiology, University of Louisville, Louisville, Kentucky, USA.

**Keywords:** bulk RNA-Seq, differential gene expression, ODC-SCI, RNA-Seq, SCI, spinal cord injury, SQLite, transcriptomics

## Abstract

Spinal cord injury (SCI) is a debilitating condition with an estimated 18,000 new cases annually in the United States. The field has accepted and adopted standardized databases such as the Open Data Commons for Spinal Cord Injury (ODC-SCI) to aid in broader analyses, but these currently lack high-throughput data despite the availability of nearly 6000 samples from over 90 studies available in the Sequence Read Archive. This limits the potential for large datasets to enhance our understanding of SCI-related mechanisms at the molecular and cellular level. Therefore, we have developed a protocol for processing RNA-Seq samples from high-throughput sequencing experiments related to SCI resulting in both raw and normalized data that can be efficiently mined for comparisons across studies, as well as homologous discovery across species. We have processed 1196 publicly available RNA-Seq samples from 50 bulk RNA-Seq studies across nine different species, resulting in an SQLite database that can be used by the SCI research community for further discovery. We provide both the database as well as a web-based front-end that can be used to query the database for genes of interest, differential gene expression, genes with high variance, and gene set enrichments.

## Introduction

Spinal cord injury (SCI) is a debilitating condition with an estimated 18,000 new cases annually in the United States, with over 80% of the cases caused by auto accident, fall, gunshot wound, motorcycle accident, or diving.^1-3^ Approximately 1.4 million Americans are living with SCI.^4^ Over the last 30 years, significant strides have been made in understanding the systems-wide pathophysiology and behavioral components affected by SCI, including axon growth,^5–10^ compensatory sprouting,^11–17^ glial cell roles,^18–22^ gut dysbiosis,^23–26^ and the role exercise plays in recovery.^27–36^ However, physical activity and training remains the only approved recovery therapy, and the complexity of molecular and cellular-level responses impedes successful translation of potential therapies.^37^ To capture and understand the heterogeneity of SCI and recovery from injury, researchers created the Open Data Commons for Spinal Cord Injury (odc-sci.org )^38^ in 2017 to provide a platform for sharing SCI-related data adhering to the FAIR principles of Findable, Accessible, Interoperable, and Resuable.^39^ As of September 28, 2022, a total of 176 complex datasets from 95 labs had been uploaded to ODC-SCI, and the structured data dictionaries that accompany each data set allow the data to be easily accessible and interoperable. While ODC-SCI contains a vast amount of data, one component that is rarely cataloged^40^ is transcriptomic data associated with RNA-Seq studies, largely because this data is generally easily accessible in resources such as the Gene Expression Omnibus (GEO)^41^ or the Sequence Read Archive (SRA).^42^ While data from SRA and GEO are accessible, they are not generally interoperable to the average researcher to perform meta-analysis comparisons across experiments.

### High-throughput datasets

The expansion in the utilization of high-throughput methodologies such as next generation sequencing, along with numerous behavioral studies, has led to the public availability of sets of data across the genome-to-phenome spectrum. Efforts to make these data usable to the larger scientific community have pushed for the data to adhere to be FAIR compliant.^39^ The issue of making these data available was first recognized in a formal manner with microarray experiments, leading to the creation of the Minimum Information About a Microarray Experiment (MIAME).^43^ The MIAME standards led to guidelines for submission of high-throughput datasets to publicly available repositories such as the GEO,^41^ the SRA,^42^ ArrayExpress,^44^ and the Database of Genotypes and Phenotypes (dbGAP).^45^ More specifically, for SCI research, standards were created for describing the minimal information about a spinal cord injury experiment (MIASCI).^46^

Since the first RNA-Seq dataset related to spinal cord injury was made available in 2013,^47^ the number of high-throughput sequencing SCI datasets (including RNA-Seq) has increased almost yearly since 2016, from five data sets submitted in 2016 to 27 submitted in 2022. More recently, efforts to make sequencing data interoperable has extended into disease-specific domains, with cancer leading the way through The Cancer Genome Atlas (TCGA)^48^ and Genomics Data Commons (GDC).^49^ Other efforts have been made to do this at tissue and gene levels through the Gene Tissue Expression (GTEx) database.^50^ However, these projects typically classify samples in a binary fashion, into healthy or diseased states. This excludes other potentially pertinent characteristics. In the case of SCI, these additional characteristics may include organism (e.g., rat, mouse, zebrafish), injury type (e.g., contusion, transection, sham), injury force (e.g., 60 kdyn, 200 kdyn, 25 g/cm), injury level (e.g., C5, 5th cervical segment; T10, 10th thoracic segment), tissue type (e.g. spinal cord tissue at epicenter, spinal cord tissue below injury, isolated astrocytes from injured spinal cord, dorsal root ganglion), and time since injury (e.g., 1 h post-injury, 24 h post-injury, 60 days post-injury). Given the shortcomings with the interoperability of SCI transcriptomic data, we have developed a pipeline for preparing publicly available datasets for comparison across studies as well as across model organisms, resulting in both an SQLite database for the raw data, as well as a web interface for integrative analysis across studies.

## Methods

### Data selection and preparation

Samples were located within the SRA^42^ using the search term “Spinal Cord Injury” OR SCI. From these, a total of 90 potential studies of interest were identified (Supplementary Table S1). Three additional SRA records related to neuropathic pain (SRP173586), nerve injury (SRP13362), and central nervous system (CNS) injury (SRP094587) were manually added, as were additional samples from the identified SRA studies that were not automatically included, due to differences in the specific search terms. A total of 5891 samples were identified from these 93 studies, falling into one of 12 categories. The number of samples is artificially inflated, due to one single-cell RNA-Seq (scRNA-Seq) study (SRP239303) where each of the 3687 cells sequenced is represented as its own sample. Those samples corresponding to an RNA-Seq assay were manually reclassified to the most appropriate assay type as RNA-Seq (bulk), scRNA-Seq, ribosomal-associated RNA-Seq (RAM-seq), and transplanted RNA-Seq. A handful of assay types were not consistently labeled and were reclassified from RNA-Seq to microRNA (miRNA-Seq) or noncoding RNA-Seq (ncRNA-Seq). The final filtered data for each of these classifications is shown in Table 1. The 50 bulk RNA-Seq studies (Table 2) were selected for further processing, representing 1196 samples from nine different species, the majority being mouse, rat, frog, and human (Table 3). RNA-Seq samples were downloaded from SRA using the sratoolkit^51^:

**Table 1.** Classification of Samples Identified

Sequencing type	Number of studies	Number of samples
Single cell RNA-Seq	15	4,062
RNA-Seq (bulk)	50	1,196
Amplicon	9	374
miRNA-Seq	6	118
Other	2	68
RAM-Seq	1	23
ncRNA-Seq	3	22
Transplanted cells	1	19
ChIP-Seq	1	4
RIP-Seq	1	4
ATAC-Seq	1	2
WGS	1	2
CLONE	1	1
Targeted capture	1	1
WXS	1	1

**Table 2.** SRA RNA-Seq Studies Included—Species Codes Are Listed in Table 3

SRA Study ID	Org	GEO ID	Study title	PubMed
SRP255811	Am		Preclinical molecular signatures of spinal cord functional restoration: optimizing the metamorphic axolotl (Ambystoma mexicanum) model in regenerative medicine	
SRP255836	Am		Identification of the molecular signatures and functional restoration of the spinal cord of metamorphic axolotl	
SRP334274	Dr	GSE182869	Next Generation Sequencing of zebrafish intraspinal serotonergic neurons in the injury segment and distal segments after spinal cord injury (zebrafish)	34876587
SRP259365	Hs	GSE149664	Generation of induced motor neurons (iMNs) from human fibroblasts facilitates locomotor recovery after spinal cord injury	32571478
SRP265127	Hs	GSE151371	Blood biomarkers for spinal cord injury (human)	33512429
SRP220569	Md		Identification of regenerative processes in neonatal spinal cord injury in the opossum (Monodelphis domestica)	
DRP003667	Mm		Genome-wide expression analysis of reactive astrocytes in the injured spinal cord at 7 days after spinal cord injury, host astrocytes in the naive spinal cord, and transplanted astrocytes in the naive spinal cord at 7 days after being transplanted	
DRP003669	Mm		Genome-wide expression analysis in the naive spinal cord and the injured spinal cord at 14 day after spinal cord injury	
SRP019916	Mm	GSE45376	RNA-Seq characterization of spinal cord injury transcriptome in acute/subacute phases: a resource for understanding the pathology at the systems level	23951329
SRP049253	Mm	GSE62698	Spinal cord injury (RNA sequencing data)	25385836
SRP067494	Mm	GSE76097	In vivo analysis of astrocyte ribosome-associated mRNA after traumatic spinal cord injury	27027288
SRP079387	Mm	GSE84737	Macrophage transcriptional profile identifies lipid catabolic pathways that can be therapeutically targeted after spinal cord injury	28130359
SRP094587	Mm	GSE90908	Characterization of meningeal type 2 innate lymphocytes and their response to CNS injury	27994070
SRP097644	Mm	GSE93976	In vivo analysis of injury sites presenting full or attenuated pericyte-derived scarring after spinal cord injury (SCI)	29502968
SRP101665	Mm	GSE96054	Time-course analysis of astrocyte-specific RNA-seq in two severities of spinal cord injury	27716282
SRP101667	Mm	GSE96055	Time-course analysis of microglia-specific RNA-seq in two severities of spinal cord injury	28420963
SRP133622	Mm	GSE111216	Mouse transcriptomics reveals extracellular matrix organization as a major pathway involved in inflammatory and neuropathic pain	30763288
SRP142367	Mm	GSE113566	Microglia and macrophages promote corralling, wound compaction and recovery in spinal cord injury via Plexin-B2	32112058
SRP173586	Mm	GSE123919	Translational profiling of dorsal root ganglia and spinal cord in a mouse model of neuropathic pain	30906902
SRP179750	Mm	GSE125176	Cellular response of mesenchymal stem cells transplanted into spinal cord injury (house mouse)	30944028
SRP201114	Mm	GSE132552	Transcriptional changes after spinal cord injury: recruitment of afferents distal to the site of injury	
SRP226573	Mm	GSE130227	Syngeneic, in contrast to allogeneic, mesenchymal stem cells have superior therapeutic potential following spinal cord injury	
SRP259320	Mm	GSE149646	Ascending dorsal column sensory neurons respond to spinal cord injury and downregulate genes related to lipid metabolism (house mouse)	33431991
SRP269775	Mm	GSE153720	Systematic analysis of purified astrocytes after spinal cord injury unveils lncRNA Zeb2os as a novel molecular target for astrogliosis [RNA-Seq] (house mouse)	33535036
SRP313384	Mm	GSE171441	Gsx1 Promotes Locomotor Functional Recovery After Spinal Cord Injury	33895323 34343529
SRP325651	Mm	GSE178930	Next Generation Sequencing Facilitates Quantitative Analysis of Wild and spinal cord injury mice (house mouse)	
SRP101364	Pm	GSE95686	RNA-Seq analysis after spinal cord injury in lamprey reveals distinct transcriptional responses during functional recovery in spinal cord and brain	29335507
SRP049326	Rn	GSE62760	T Cell Deficiency in Spinal Cord Injury: Altered Locomotor Recovery and Whole-Genome Transcriptional Analysis	26546062
SRP073355	Rn		Transcriptome of Sprague-Dawley Rats – spinal cord contusion	
SRP096190	Rn	GSE93249	RNA-Seq analysis of coding and long non-coding RNAs in the sub-chronic and chronic stages of spinal cord injury	28106101
SRP131816	Rn	GSE109902	Transcriptional screen in the target region of sprouting hindlimb corticospinal fibers after thoracic spinal cord injury in rats (Norway rat)	
SRP149309	Rn	GSE115067	Integrated systems analysis reveals conserved gene networks underlying response to spinal cord injury (Norway rat)	30277459
SRP166392	Rn		Following 6 days differentiation of neural stem cells in vitro	
SRP176640	Rn	GSE124819	Activity-induced changes in the liver transcriptome after chronic spinal cord injury	31197156 32226812
SRP179652	Rn	GSE125134	Analysis of possible mechanisms behind functional recovery following neural progenitor cell transplantation into spinal cord injury. (Norway rat)	31031190
SRP181953	Rn	GSE125630	Transcriptome of dorsal root ganglia caudal to a spinal cord injury with modulated behavioral activity	31175296 32226812
SRP192162	Rn	GSE129694	Transcriptional changes in soleus muscle for rats exposed to different activities after contusion injury to the spinal cord and transcriptional changes in soleus muscle with complete spinal cord transection injury	32226812
SRP202013	Rn	GSE133093	Brainstem control of transcription after spinal cord injury (SCI)	31803022
SRP213314	Rn		Transcriptomic analysis of knockdown of ?-synuclein after T3 spinal cord injury in rats	
SRP216808	Rn	GSE135080	Novel drug-like RAR-Beta agonist induces BRCA1 to prevent neuropathic pain (Norway rat)	31726373
SRP224959	Rn	GSE138637	Genome wide analysis of thoracic spinal cord at 5 days after T9 hemisection injury.	
SRP273616	Rn		Ketogenic diet-mediated steroid metabolism reprogramming improves the immune microenvironment and myelin growth of spinal cord injury rats through gene analysis and co-expression network analysis	
SRP275629	Rn	GSE155610	Transcriptome of Subcortical White Matter and Spinal Cord After Spinal Injury and Cortical Stimulation	34267212
SRP279076	Rn	GSE156999	Circular RNAs expression profiles and potential key molecules incompletely transected spinal cord injury	
SRP311591	Rn		Sequencing for spinal cord injury	
SRP082501	Ts		Pool of tissues of Trachemys scripta elegans	
DRP006873	Xl		RNA-seq analysis after spinal cord injury in Xenopus laevis	
SRP222957	Xl	GSE137844	Comparative Gene Expression Profiling between Xenopus Optic Nerve and Spinal Cord Injury to Identify Genes Involved in Successful Regeneration of Vertebrate CNS Axons	32758133 34979916
SRP300206	Xl	GSE164204	Cellular response to spinal cord injury in regenerative and non-regenerative stages in Xenopus laevis	33526076
SRP302901	Xl	GSE165343	High expression profiling analysis of the early response to spinal cord injury identified a key role for mTORC1 signaling	34686684

**Table 3.** Samples by Species

Species name	Common name	Species code	Number of RNA-Seq samples
*Mus musculus*	House mouse	Mm	569
*Rattus norvegicus*	Norway rat	Rn	297
*Xenopus laevis*	African clawed frog	Xl	176
*Homo sapiens*	Human	Hs	65
*Mondelphis domestica*	Gray short-tailed opossum	Md	42
*Petromyzon marinus*	Sea lamprey	Dm	22
*Danio rerio*	Zebrafish	Dr	11
*Ambystoma mexicanum*	Axolotl	Am	8
*Trachemys scripta elegans*	Red-eared slider turtle	Ts	6




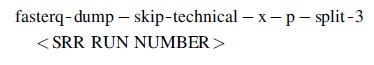




Eleven rat samples were omitted (SRR13562711-SRR13562722) due to issues with obtaining the samples with sratoolkit. Samples were then processed for quality assurance and quality control (QA/QC) using fastQC.^52^

Sequencing reads were aligned to the respective reference genomes guided by known transcriptomes (Table 4) using STAR.^53^ Raw read counts were determined using the featureCounts R package.^54^ Counts were extracted using three separate stranded settings, including –s 0 (unstranded), -s 1 (forward stranded), and –s 2 (reverse stranded). This step was performed to infer the most likely strandedness of the original library construction kit. Strandedness was also calculated using the infer_experiment.py script from the RSeqC package.^55^ The resulting feature counts were reformatted to the same format of htSeqCount^56^ using a custom script. The count information was then parsed to create a tab-delimited file of raw gene counts and TPM (transcripts per million) normalized counts.^57^ Since TPM normalizes to both the library size and the transcript size, we utilized the R GenomicFeatures package^58^ to determine gene lengths. The exception to this was the axolotl data, where a known transcriptome description is not currently available. In this case, those samples were constructed into *de novo* transcriptomes using Trinity.^59^

**Table 4.** Reference Genomes and Gene Description Files Used

Organism	Reference genome (accession)	GTF
*Mus musculus*	GRCm38.p6 (mm10)	Ensembl v101 (Mus_musculus.GRCm38.101.gtf )
*Rattus norvegicus*	Rnor_6.0 (rn6)	Ensembl v101 (Rattus_norvegicus.Rnor_6.0.101.gtf )
*Xenopus laevis*	Xenla10.1	Genbank (GCF_017654675.1_Xenopus_laevis_v10.1_genomic.gtf )
*Homo sapiens*	GRC38 (hg38) (GRC38.d1.vd1.fa)	Gencode v22 (gencode.v22.annotation.gtf )
*Mondelphis domestica*	ASM229v1	Ensembl v104 (Monodelphis_domestica.ASM29v1.104.gtf )
*Petromyzon marinus*	kPetMar1 (GCF_010993605.1)	Genbank (GCF_0100993605.1_kPetMar1.pri_genomic.gtf )
*Danio rerio*	GRCz11	Ensembl v105 (Danio_rerio_GRCz11.105.chr.gtf )
*Trachemys scripta elegans*	CAS_Tse_1.0 (GCF_013100865.1)	Genbank (GCF_013100865.1_CAS_Tse_1.0_genomic.gtf )
*Ambystoma mexicanum*	AmbMex60DD (GCA_002915635.3)	(Transcriptome constructed from Trinity)

### SQLite database construction

Tab-delimited files representing data for 10 SQLite tables were constructed, and then parsed into the SQLite database. Each table was connected to other tables via foreign keys, including the organism, gene identifier, SRA study identifier, and SRA sample identifier.

### Data exploration

When a user chooses the data exploration option (described below), the samples of interest are filtered by species using the raw sequencing reads determined by featureCounts. If samples from at least one of the groups mouse and human, mouse and rat, human and rat, or mouse, human, and rat are selected, then the genes are filtered for homologous genes across species using the NCBI Homologene identifiers.^60^ Differential gene expression is calculated for the samples within each species individually, as well as for homologous genes across species using DESeq2.^61^ The resulting data is sorted by q-value (Storey's false discovery rate correction) from smallest to largest and parsed for up-regulated genes (those with a positive logFC) and down-regulated genes (those with a negative logFC). Heatmaps for the top 250 differentially expressed and top 250 variance genes are constructed, using a gene-wise z-score. Enriched GeneOntology Biological Processes (GO:BP)^62^ and KEGG pathway^63^ are determined using ClusterProfiler.^64^

## Results

### RNA-Seq data

**FIG. 1.**
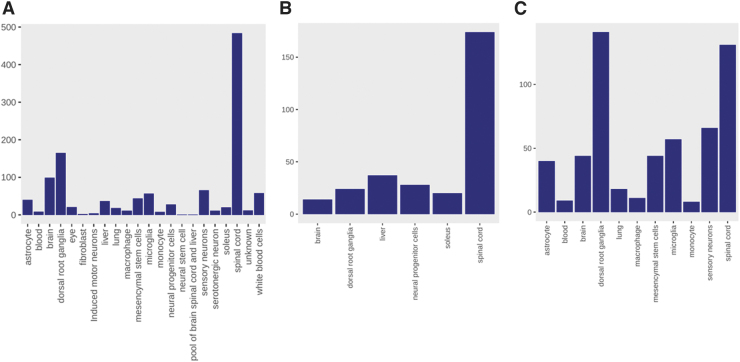
Tissue types studied in filtered RNA-Seq experiments. Shown are **(A)** the tissue types for all samples; **(B)** tissue types for rat experiments; and **(C)** tissue types for mouse experiments.

A total of 1196 samples from 50 bulk RNA-Seq studies were processed for inclusion in the SQLite database, with the majority originating from either spinal cord or dorsal root ganglion tissue (Fig. 1). The injury site for these studies was varied, with the most common location thoracic—in particular, thoracic vertebrae 8 (T8) and T10 (Supplementary Fig. S1). The time since injury was varied, with the majority occurring within the first 2 weeks, although a number of studies extended the time, all the way up to 24 weeks (Supplementary Fig. S2). Over 30 unique injury types are represented (Supplementary Fig. S3), with the most frequent being complete transection, contusion, and various controls, including sham and laminectomy. In many cases, the exact control type could not be extracted from the GEO entry and any associated publication, and is therefore listed as uninjured or control. The number of human samples is limited, in part due to the controlled nature of human samples and in part due to the scarcity of tissue (spinal cord and dorsal root ganglion [DRG]) from individuals with spinal cord injuries. A search of dbGaP^65^ results in only one study with spinal cord injury samples, with four studies with spinal cord tissue and three with DRG, mainly in the context of pain.

### SQLite database

**FIG. 2.**
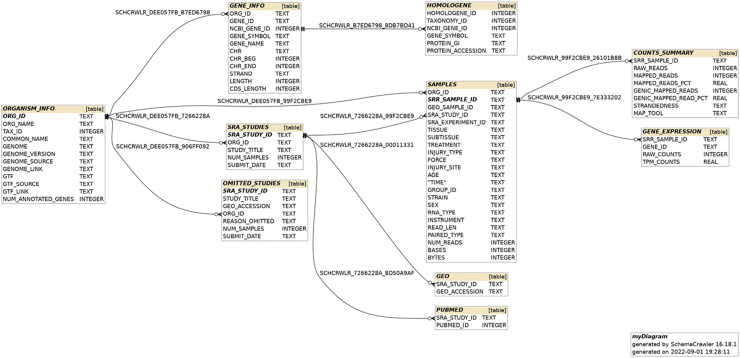
Entity-relationship (ER) model for the SQLite database.

**FIG. 3.**
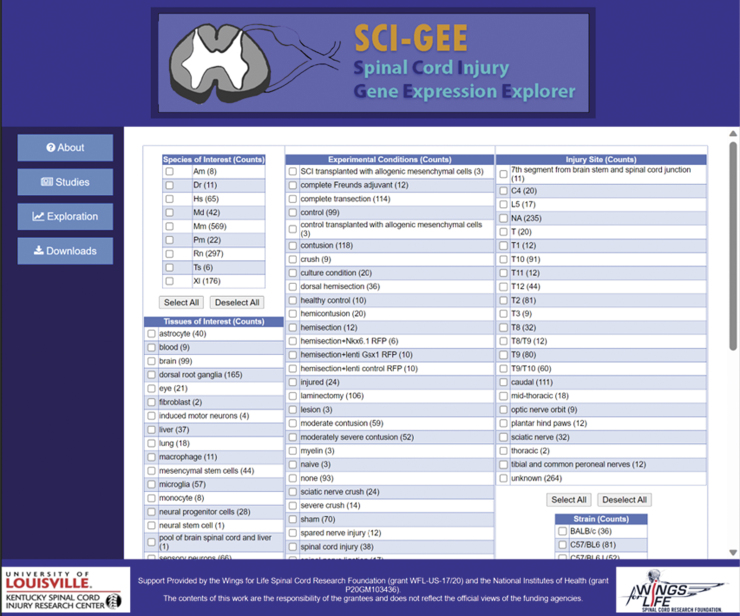
Data exploration page. SCI-GEE users are presented with choices for species of interest, tissues of interest, injury of interest, and injury site of interest.

After the studies and their corresponding samples were processed, the resulting information was structured into an SQLite relational database consisting of 10 tables, as shown in the entity-relationship (ER) diagram in Figure 2. The total database size is just under 2.5 Gb. Web front-end. Utilization of the SQLite database was performed using a web-based application for querying the data. Presented on the web page is information about the constructed database (Supplementary Fig. S4), links and summaries for included studies (Supplementary Fig. S5), downloadable files (Supplementary Fig. S6), and database exploration (Fig. 3).

### Data exploration

The user begins a data exploration by first selecting the organisms, tissues, injury types, injury locations, sex, strain, and specific genes of interest. They are then presented with the resulting sample groups that can be selected for a pairwise analysis (Supplementary Fig. S7). Once the differential expression analysis is complete, the user is returned a set of results, including the top 25 up- and down-regulated genes (Supplementary Fig. S8), heatmaps for the top 250 differentially expressed genes (Supplementary Fig. S9) and genes with the highest variance, principal component analysis (PCA) plot, volcano plot, heatmap for genes of interest, and GO:BP and KEGG pathway enriched categories.

## Discussion

### Case usage studies

**FIG. 4.**
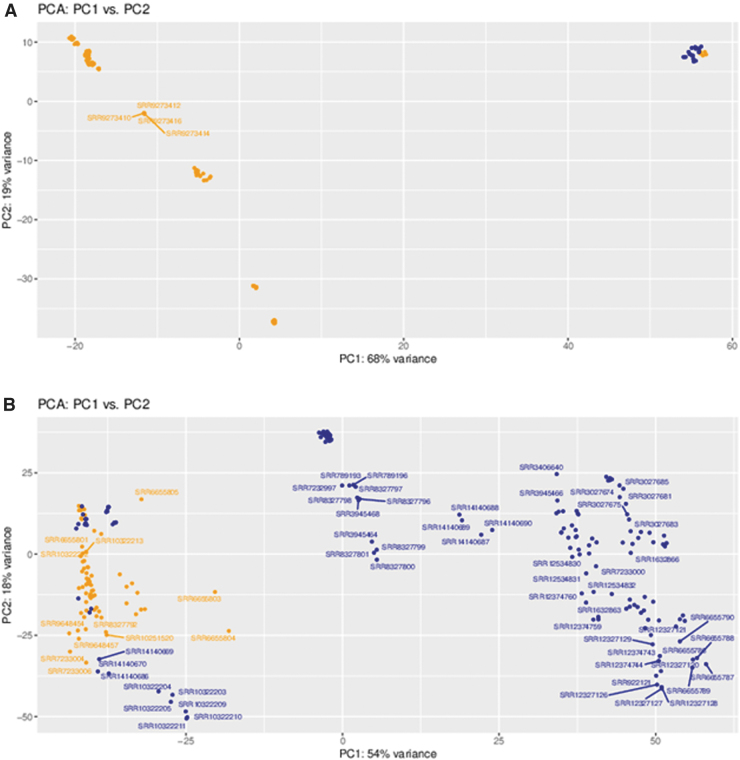
Principal component analysis (PCA) based on gene expression. Shown are **(A)** PCA plot for dorsal root ganglion (DRG) samples and **(B)** PCA plot for spinal cord tissues. Control samples are shown in orange and injured samples are shown in blue.

Demonstration of the utility of SCI-GEE was performed with two usage studies in mouse and rat: 1) analysis of DRG in spinal cord injured versus uninjured samples across all available injury sites; and 2) spinal cord tissue in spinal cord injured versus uninjured samples. Mouse and rat were selected since they have the highest representation in the database. For both studies, results were available for three groups, including mouse and rat homologs; mouse genes only; and rat genes only. In the case of mouse and rat homologs, a total of 16,529 genes were compared based on shared homologene IDs,^60^ while the mouse samples were compared using 55,487 genes (including both protein coding and noncoding genes), and 32,883 rat genes were utilized for that comparison. While the data analyzed in SCI-GEE is available for download and utilization in other analytical workflows of interest, the results presented here are fully contained within the website functionality. For both tissue sets, the control and injured samples separated as illustrated by the PCA plots for DRG (Fig. 4A) and spinal cord (Fig. 4B), although a high degree of variance was found in the injured samples due to variability in other experimental factors, including injury type, severity, and time since injury.

### DRG tissue analysis

For the DRG tissue dataset, two groups were created across mouse and rat: control samples (including both naïve and sham models) and spinal cord injured samples (Supplementary Table S2). The spinal cord injuries were from a variety of models, including hemisection, transection, and contusion. One of the meta-analysis capabilities of SCI-GEE is the ability to perform cross-species analysis across human, mouse, and rat by looking at the expression of common homologs across species. In these usage cases, shared homologene IDs across mouse and rat were examined.

The top 25 up-regulated genes are shown in Supplementary Table S3, while the top 25 down-regulated genes are shown in Supplementary Table S4. Included in the up-regulated genes in the DRG for the mouse and rat meta-analysis are GADD45A, which is involved in cell cycle and neuronal cell death^66^ and may act in a neuroprotective fashion^67-69^; GPR151, which is involved in neuropathic pain, and may promote axon regeneration^70,71^; FLRT3, which promotes neurite outgrowth^72-74^; CRLF1, which forms complexes with neurotrophic factors to promote survival of neuronal cells^75^; and the neuropeptide GAL.^76,77^ At the top of the down-regulated genes are two leucine zipper proteins (FOSB and FOS), two zinc fingers (EGR1 and ZFP36), and CYR61. These all play roles in regulating cell proliferation and apoptosis, indicating that regulatory machinery is turned off in response to spinal cord injury. In the DRG for mouse only, the top 25 up-regulated genes are shown in Supplementary Table S5, and the top 25 down-regulated are shown in Supplementary Table S6.

At the top of the up-regulated genes are GADD45A, FST, GPR151, IGFN1, and TES. The up-regulation of FST (follistatin) and TES (testin LIM domain protein) may be indicative of sex-specific differences in the makeup of the mouse samples, while GPR151 is known to modulate neuropathic pain^78,79^ and IGFN1 is involved in synapse assembly.^80^ The top mouse down-regulated genes are similar to those found for mouse and rat homologs. The top 25 up-regulated genes in DRG for the rat only are shown in Supplementary Table S7, and the top 25 down-regulated genes are shown in Supplementary Table S8. Among the up-regulated genes are several members of the Hox gene family (Hoxc11, Hoxd10, and Hoxd11), which are important for motor neuron patterning,^81–84^ as well as peripheral myelin protein 2 (Pmp2). The down-regulated genes include Usp5, which modulates neuropathic and inflammatory pain^85^; Tuba1a, which is necessary for central nervous system development and regeneration^86^; Csf1r, which promotes microglial proliferation^87,88^; and Slc39a6, which is found in reactive astrocytes.^89^

**FIG. 5.**
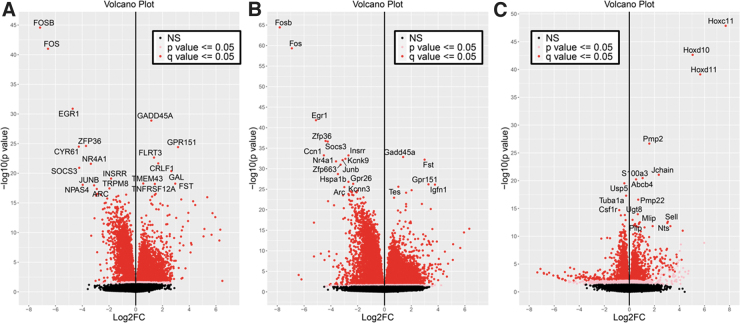
Volcano plots shown for dorsal root ganglion (DRG) samples. Shown are **(A)** meta-analysis of mouse and rat homologs combined, **(B)** mouse genes, and **(C)** rat genes.

For each of the comparisons (both, mouse, and rat, respectively), volcano plots were generated to show the overall pattern of expression, including the fold-change across the x-axis and the q-value significance across the y-axis (Fig. 5A-C), which illustrates a relative even distribution of the data. Heatmaps were generated showing the top differentially expressed genes (Supplementary Fig. S10A-C), with the genes listed across the rows, and the samples across the columns.

### DRG enrichment analysis

**FIG. 6.**
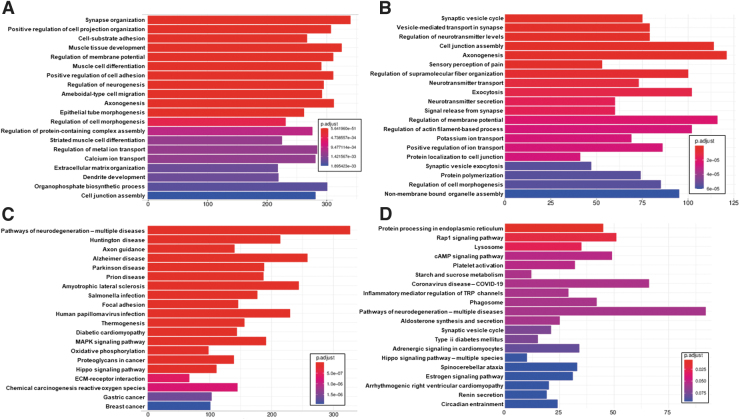
Top 20 clusterProfiler enrichments for dorsal root ganglion (DRG) injury. Included are top 20 GO:BP enrichments for DRG injury vs. control comparison for **(A)** mouse samples and **(B)** rat samples and top 20 KEGG metabolic pathway enrichments for **(C)** mouse samples and **(D)** rat samples.

Differentially expressed genes from the mouse and rat comparisons were used as input into ClusterProfiler^64^ for analysis of enriched GO:BP^62^ and KEGG Metabolic Pathways.^63^ The top 20 GO:BP enrichments are shown in Figure 6A and 6B for mouse and rat respectively, while the top 20 KEGG enrichments are shown in Figure 6C and 6D. For the mouse differentially expressed genes, several of the top GO:BP annotations are associated with nervous system development, including synapse organization, regulation of neurogenesis, axonogenesis, epithelial tube morphogenesis, and dendrite development, potentially indicating responses for regeneration and/or collateral sprouting. Others are associated with muscular development, including muscle tissue development, muscle cell differentiation, and striated muscle cell differentiation. A third cluster is related to cell signaling and adhesion, including cell-substrate adhesion, positive regulation of cell adhesion, regulation of metal ion transport, calcium ion transport, and cell junction assembly.

Results are similar for the rat, including synaptic vesicle cycle, vesicle-mediated transport in synapse, axonogenesis, regulation of neurotransmitter levels, sensory perception of pain, neurotransmitter transport, neurotransmitter secretion, signal release from synapse, regulation of membrane potential, potassium ion transport, positive regulation of ion transport, protein localization to cell junction, and synaptic vesicle exocytosis. The KEGG pathway enrichment results are less clear, but include pathways of neurodegeneration, axon guidance and synaptic vesicle cycle, along with a number of seemingly unrelated pathways, many of which are tied to proinflammatory responses.

### Spinal cord tissue analysis

In the case of the spinal cord tissues, two groups were created across mouse and rat: control samples (including both naïve and sham models) and spinal cord injured samples (Supplementary Table S9). The spinal cord injuries were from a variety of models, including hemisection, transection, hemicontusion, and contusion. Among the top up-regulated differentially expressed genes for the meta-analysis across mouse and rat homologs (Supplementary Table S10) are SIGLEC1 (CD169), which signals an increase in metallophilic macrophages and an increased immune response^90^; GPNMB, a glioma-associated glycoprotein^91^; MPEG1, a macrophage/microglia marker gene^92,93^; CCL13 which is produced from M2 macrophages^94^; and CD68, a marker for phagocytic microglia.^95^ Down-regulated genes (Supplementary Table S11) include HMGCS1, HMGCR MSMO1 and IDI1, which are involved in cholesterol metabolism, and whose down-regulation may prevent demyelination.^96,97^ The mouse-only study shows similar results to the mouse and rat homologs, with Gpnmb, Ccl2, Bst2, Lyz2, and Trem2 among the top up-regulated genes (Supplementary Table S12). Ccl2 is a cytokine functioning in inflammation and pain following spinal cord injury,^98^ while Bst2 is a neuroinflammation biomarker.^99^ Lyz2 is expressed in reactive microglia and macrophages,^100^ and Trem2 elicits a proinflammatory response in microglia.^101^ Down-regulated genes include Cltrn, Aldob, Rapgef5, Mep1a, and Slc22a12 (Supplementary Table S13). Aldob has a role in glycolysis and glucogenesis,^102^ while Rapgef5 is involved in signal transduction with the Ras pathway.^103^

Up-regulated genes for the rat-only spinal cord tissue includes Siglec1, Lilrb3, Gpnmb, Clec7a, and Mmp12 (Supplementary Table S14), similar to the results for the mouse and rat homologs. Ablation of Lilrb3 has been shown to promote neurite outgrowth,^104^ while Clec7a is a marker of actively proliferating microglia^105^ and Mmp12 is involved in myelination. The top down-regulated rat genes include a number of genes with unknown function (Supplementary Table S15). Among those that have associated functions are Hmgcr, Mff, and Msmo1. As previously mentioned, Hmgcr and Msmo1 function in cholesterol metabolism, while Mff is associated with lengthening neuron life.^106^ Volcano plots and heatmaps are provided in Supplementary Figure S11A and S11B, and Supplementary Figure S12A and S12B, respectively.

### Spinal cord enrichment analysis

**FIG. 7.**
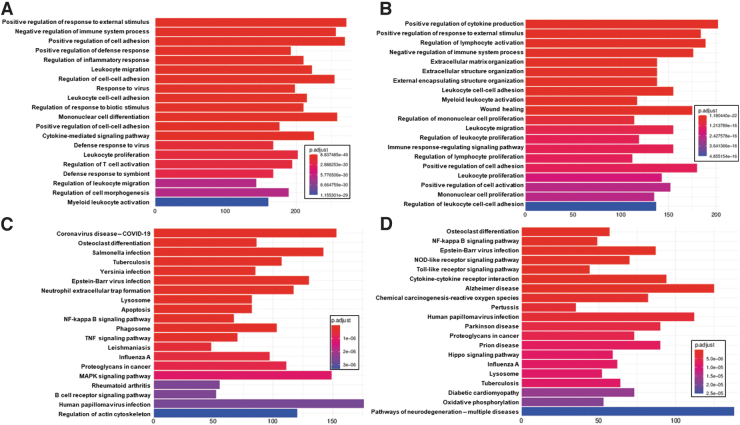
Top 20 clusterProfiler enrichments for spinal cord injury. Included are top 20 GO:BP enrichments for spinal cord injury vs. control comparison for **(A)** mouse samples and **(B)** rat samples and top 20 KEGG metabolic pathway enrichments for **(C)** mouse samples and **(D)** rat samples.

Among the top 20 GO:BP enrichments (Fig 7A, 7B) are a number of proinflammatory annotations, including negative regulation of immune system process, regulation of inflammatory response, leukocyte migration, leukocyte cell-cell adhesion, cytokine-mediated signaling pathway, leukocyte proliferation, regulation of T cell activation, regulation of leukocyte migration, and myeloid leukocyte activation. Enriched KEGG metabolic pathways (Fig. 7C, 7D) include a number of signaling pathways (NF-kappa B signaling pathway, TNF signaling pathway, MAPK signaling pathway, B cell receptor signaling pathway, NOD-like receptor signaling pathway, Toll-like receptor signaling pathway, cytokine-cytokine receptor interaction, and Hippo signaling pathway, indicating that spinal cord injury leads to a number of signaling cascades in the spinal cord itself, consistent with previous independent findings,^107–111^ as well as within the studies whose samples are included in this case study.^112–118^

### Single cell transcriptomics

More recent transcriptome work in the SCI field has included studies utilizing single cell RNA-Seq (scRNA-Seq),^100,119-125^ including a recent atlas of spinal cord injury in mice.^123^ Due to the complexities of scRNA-Seq data, including cell typing and the disparity between limited sample numbers and large numbers of cells per sample, as well as a different modality for viewing scRNA-Seq data, we chose not to include those data at this time to focus on the more broadly available bulk RNA-Seq datasets. Over time, we anticipate that more scRNA-Seq datasets will be generated for SCI as costs come down. When that happens, we plan to develop a parallel method for integrating scRNA-Seq data. Such a resource would allow for more resolution at a single cell level, providing for an atlas of transcriptional changes for cells affected by SCI.

## Conclusions

Our approach for preparing bulk RNA-Seq data related to SCI allows for greater interoperability across datasets. This in turn enables for meta-analyses both within and across species. By providing the data in an SQLite format, potential users are able to either utilize our web interface for exploring the available data, or develop their own analysis pipelines the utilizes the prepared data. We hope this work will eventually be incorporated into ODC-SCI data types, allowing for even greater integration along the genome-to-phenome modalities.

## Transparency, Rigor, and Reproducibility Summary

All datasets utilized in this research are publicly available in the Sequence Read Archive and can be accessed using the SRA IDs available in Supplementary Table S2. The complete details of the data processing are provided in the Methods section. All raw and processed transcript count data is provided at the website: http://162.215.210.70/∼tracks/SCI-GEE .

## References

[1] National Spinal Cord Injury Statistical Center. Spinal Cord Injury Model Systems 2021 Annual Report. Birmingham, AL; 2021. Available from: https://www.nscisc.uab.edu/PublicDocuments/AR2021_public%20version.pdf [Last accessed June 20, 2023].

[2] National Spinal Cord Injury Statistical Center. Frequently Asked Questions. Birmingham, AL; 2022. Available from: https://www.nscisc.uab.edu/Public_Pages/FAQ [Last accessed September 28, 2022].

[3] Jain NB, Ayers GD, Peterson EN, et al. Traumatic spinal cord injury in the United States, 1993-2012. JAMA 2015;313(22):2236–2243; doi: 10.1001/jama.2015.6250 26057284 PMC4712685

[4] Christopher and Dana Reeve Foundation. Stats about paralysis. Short Hills, NJ; 2022. Available from: https://www.christopherreeve.org/living-with-paralysis/stats-about-paralysis [Last accessed September 28, 2022].

[5] Anderson MA, O'Shea TM, Burda JE, et al. Required growth facilitators propel axon regeneration across complete spinal cord injury. Nature 2018;561(7723):396–400; doi: 10.1038/s41586-018-0467-6 30158698 PMC6151128

[6] Bregman BS, McAtee M, Dai HN, et al. Neurotrophic factors increase axonal growth after spinal cord injury and transplantation in the adult rat. Exp Neurol 1997;148(2):475–494; doi: 10.1006/exnr.1997.6705 9417827

[7] Hellal F, Hurtado A, Ruschel J, et al. Microtubule stabilization reduces scarring and causes axon regeneration after spinal cord injury. Science 2011;331(6019):928–931; doi: 10.1126/science.1201148 21273450 PMC3330754

[8] Lu P, Jones LL, Snyder EY, et al. Neural stem cells constitutively secrete neurotrophic factors and promote extensive host axonal growth after spinal cord injury. Exp Neurol 2003;181(2):115–129; doi: 10.1016/s0014-4886(03)00037-2 12781986

[9] Lu P, Jones LL, Tuszynski MH. BDNF-expressing marrow stromal cells support extensive axonal growth at sites of spinal cord injury. Exp Neurol 2005;191(2):344–360; doi: 10.1016/j.expneurol.2004.09.018 15649491

[10] Lu P, Woodruff G, Wang Y, et al. Long-distance axonal growth from human induced pluripotent stem cells after spinal cord injury. Neuron 2014;83(4):789-96; doi: 10.1016/j.neuron.2014.07.014 25123310 PMC4144679

[11] Barritt AW, Davies M, Marchand F, et al. Chondroitinase ABC promotes sprouting of intact and injured spinal systems after spinal cord injury. J Neurosci 2006;26(42):10856–10867; doi: 10.1523/JNEUROSCI.2980-06.2006 17050723 PMC3339436

[12] Fouad K, Pedersen V, Schwab ME, et al. Cervical sprouting of corticospinal fibers after thoracic spinal cord injury accompanies shifts in evoked motor responses. Curr Biol 2001;11(22):1766–1770; doi: 10.1016/s0960-9822(01)00535-8 11719218

[13] Lee JK, Geoffroy CG, Chan AF, et al. Assessing spinal axon regeneration and sprouting in Nogo-, MAG-, and OMgp-deficient mice. Neuron 2010;66(5):663–670; doi: 10.1016/j.neuron.2010.05.002 20547125 PMC2896331

[14] Maier IC, Schwab ME. Sprouting, regeneration and circuit formation in the injured spinal cord: factors and activity. Philos Trans R Soc Lond B Biol Sci 2006;361(1473):1611–1634; doi: 10.1098/rstb.2006.1890 16939978 PMC1664674

[15] Raineteau O, Schwab ME. Plasticity of motor systems after incomplete spinal cord injury. Nat Rev Neurosci 2001;2(4):263–273; doi: 10.1038/35067570 11283749

[16] Schwab ME. Repairing the injured spinal cord. Science 2002;295(5557):1029–1031; doi: 10.1126/science.1067840 11834824

[17] Weidner N, Ner A, Salimi N, et al. Spontaneous corticospinal axonal plasticity and functional recovery after adult central nervous system injury. Proc Natl Acad Sci U S A 2001;98(6):3513–3518; doi: 10.1073/pnas.051626798 11248109 PMC30684

[18] Barnabe-Heider F, Goritz C, Sabelstrom H, et al. Origin of new glial cells in intact and injured adult spinal cord. Cell Stem Cell 2010;7(4):470–482; doi: 10.1016/j.stem.2010.07.014 20887953

[19] Gaudet AD, Fonken LK. Glial Cells Shape Pathology and Repair After Spinal Cord Injury. Neurotherapeutics 2018;15(3):554–577; doi: 10.1007/s13311-018-0630-7 29728852 PMC6095774

[20] Gwak YS, Kang J, Unabia GC, et al. Spatial and temporal activation of spinal glial cells: role of gliopathy in central neuropathic pain following spinal cord injury in rats. Exp Neurol 2012;234(2):362–372; doi: 10.1016/j.expneurol.2011.10.010 22036747 PMC3303938

[21] Kim D, Kim MA, Cho IH, et al. A critical role of toll-like receptor 2 in nerve injury-induced spinal cord glial cell activation and pain hypersensitivity. J Biol Chem 2007;282(20):14975–14983; doi: 10.1074/jbc.M607277200 17355971

[22] Liu XZ, Xu XM, Hu R, et al. Neuronal and glial apoptosis after traumatic spinal cord injury. J Neurosci 1997;17(14):5395–5406; doi: 10.1523/JNEUROSCI.17-14-05395.1997 9204923 PMC6793816

[23] Gungor B, Adiguzel E, Gursel I, et al. Intestinal microbiota in patients with spinal cord injury. PLoS One 2016;11(1):e0145878; doi: 10.1371/journal.pone.0145878 26752409 PMC4709077

[24] Kigerl KA, Hall JC, Wang L, et al. Gut dysbiosis impairs recovery after spinal cord injury. J Exp Med 2016;213(12):2603-2620; doi: 10.1084/jem.20151345 27810921 PMC5110012

[25] Kigerl KA, Mostacada K, Popovich PG. Gut Microbiota Are Disease-Modifying Factors After Traumatic Spinal Cord Injury. Neurotherapeutics 2018;15(1):60–67; doi: 10.1007/s13311-017-0583-2 29101668 PMC5794696

[26] Zhang C, Zhang W, Zhang J, et al. Gut microbiota dysbiosis in male patients with chronic traumatic complete spinal cord injury. J Transl Med 2018;16(1):353; doi: 10.1186/s12967-018-1735-9 30545398 PMC6293533

[27] Battistuzzo CR, Callister RJ, Callister R, et al. A systematic review of exercise training to promote locomotor recovery in animal models of spinal cord injury. J Neurotrauma 2012;29(8):1600–1613; doi: 10.1089/neu.2011.2199 22401139 PMC3353762

[28] Chariker JH, Ohri SS, Gomes C, et al. Activity/exercise-induced changes in the liver transcriptome after chronic spinal cord injury. Sci Data 2019;6(1):88; doi: 10.1038/s41597-019-0087-5 31197156 PMC6565704

[29] Dupont-Versteegden EE, Houle JD, Dennis RA, et al. Exercise-induced gene expression in soleus muscle is dependent on time after spinal cord injury in rats. Muscle Nerve 2004;29(1):73–81; doi: 10.1002/mus.10511 14694501

[30] Engesser-Cesar C, Anderson AJ, Basso DM, et al. Voluntary wheel running improves recovery from a moderate spinal cord injury. J Neurotrauma 2005;22(1):157–171; doi: 10.1089/neu.2005.22.157 15665610

[31] Fu J, Wang H, Deng L, et al. Exercise Training Promotes Functional Recovery after Spinal Cord Injury. Neural Plast 2016;2016:4039580; doi: 10.1155/2016/4039580 28050288 PMC5168470

[32] Hutchinson KJ, Gomez-Pinilla F, Crowe MJ, et al. Three exercise paradigms differentially improve sensory recovery after spinal cord contusion in rats. Brain 2004;127(Pt 6):1403–1414; doi: 10.1093/brain/awh160 15069022

[33] Sandrow-Feinberg HR, Houle JD. Exercise after spinal cord injury as an agent for neuroprotection, regeneration and rehabilitation. Brain Res 2015;1619:12–21; doi: 10.1016/j.brainres.2015.03.052 25866284 PMC4540698

[34] Smith RR, Brown EH, Shum-Siu A, et al. Swim Training Initiated Acutely after Spinal Cord Injury Is Ineffective and Induces Extravasation In and Around the Epicenter. J Neurotrauma 2009;26(7):1017–1027; doi: 10.1089/neu.2008.0829 19331515 PMC2848951

[35] Smith RR, Shum-Siu A, Baltzley R, et al. Effects of swimming on functional recovery after incomplete spinal cord injury in rats. J Neurotrauma 2006;23(6):908–919; doi: 10.1089/neu.2006.23.908 16774475 PMC2831776

[36] Van Meeteren NL, Eggers R, Lankhorst AJ, et al. Locomotor recovery after spinal cord contusion injury in rats is improved by spontaneous exercise. J Neurotrauma 2003;20(10):1029–1037; doi: 10.1089/089771503770195876 14588119

[37] Anjum A, Yazid MD, Fauzi Daud M, et al. Spinal Cord Injury: Pathophysiology, multimolecular interactions, and underlying recovery mechanisms. Int J Mol Sci 2020;21(20):7533; doi: 10.3390/ijms21207533 33066029 PMC7589539

[38] Callahan A, Anderson KD, Beattie MS, et al. Developing a data sharing community for spinal cord injury research. Exp Neurol 2017;295:135–143; doi: 10.1016/j.expneurol.2017.05.012 28576567 PMC6448396

[39] Wilkinson MD, Dumontier M, Aalbersberg IJ, et al. The FAIR Guiding Principles for scientific data management and stewardship. Sci Data 2016;3(1):160018; doi: 10.1038/sdata.2016.18 26978244 PMC4792175

[40] Kyritsis N, Torres-Espin A, Schupp PG, et al. ODC-SCI. Acute blood RNA sequencing from SCI human patients. 2021. Available from: https://odc-sci.org/data/405 [Last accessed June 20, 2023]. doi: 10.34945/F5QC7J

[41] Edgar R, Domrachev M, Lash AE. Gene Expression Omnibus: NCBI gene expression and hybridization array data repository. Nucleic Acids Res 2002;30(1):207–210, doi: 10.1093/nar/30.1.207 11752295 PMC99122

[42] Leinonen R, Sugawara H, Shumway M, et al. The Sequence Read Archive. Nucleic Acids Res 2011;39(Database issue):D19–2; doi: 10.1093/nar/gkq1019 21062823 PMC3013647

[43] Brazma A, Hingamp P, Quackenbush J, et al. Minimum information about a microarray experiment (MIAME) —toward standards for microarray data. Nat Genet 2001;29(4):365–371; doi: 10.1038/ng1201-365 11726920

[44] Brazma A, Parkinson H, Sarkans U, et al. ArrayExpress—a public repository for microarray gene expression data at the EBI. Nucleic Acids Res 2003;31(1):68–71; doi: 10.1093/nar/gkg091 12519949 PMC165538

[45] Mailman MD, Feolo M, Jin Y, et al. The NCBI dbGaP database of genotypes and phenotypes. Nat Genet 2007;39(10):1181–1186; doi: 10.1038/ng1007-1181 17898773 PMC2031016

[46] Lemmon VP, Ferguson AR, Popovich PG, et al. Minimum information about a spinal cord injury experiment: a proposed reporting standard for spinal cord injury experiments. J Neurotrauma 2014;31(15):1354–1361; doi: 10.1089/neu.2014.3400 24870067 PMC4120647

[47] Chen K, Deng S, Lu H, et al. RNA-Seq characterization of spinal cord injury transcriptome in acute/subacute phases: a resource for understanding the pathology at the systems level. PloS One 2013;8(8):e72567; doi: 10.1371/journal.pone.0072567 23951329 PMC3739761

[48] Weinstein JN, Collisson EA, Mills GB, et al. The cancer genome atlas pan-cancer analysis project. Nat Genet 2013;45(10):1113–1120; doi: 10.1038/ng.2764 24071849 PMC3919969

[49] Grossman RL, Heath AP, Ferretti V, et al. Toward a shared vision for cancer genomic data. New Engl J Med 2016;375(12):1109–1112; doi: 10.1056/NEJMp1607591. 27653561 PMC6309165

[50] Lonsdale J, Thomas J, Salvatore M, et al. The genotype-tissue expression (GTEx) project. Nat Genet 2013;45(6):580–585; doi: 10.1038/ng.2653 23715323 PMC4010069

[51] Sayers EW, O'Sullivan C, Karsch-Mizrachi I. Using GenBank and SRA. In: Plant Bioinformatics. Methods in Molecular Biology, vol 2443. (Edwards D. ed.). Humana, New York; 2022; pp. 1–25. 10.1007/978-1-0716-2067-0_1 35037198

[52] Andrews S. Babraham Bioinformatics. FastQC: a quality control tool for high throughput sequence data. Cambridge, UK, 2010. Available from: https://www.bioinformatics.babraham.ac.uk/projects/fastqc [Last accessed June 20, 2023].

[53] Dobin A, Davis CA, Schlesinger F, et al. STAR: ultrafast universal RNA-Seq aligner. Bioinformatics 2013;29(1):15–21; doi: 10.1093/bioinformatics/bts635 23104886 PMC3530905

[54] Liao Y, Smyth GK, Shi W. featureCounts: an efficient general purpose program for assigning sequence reads to genomic features. Bioinformatics 2014;30(7):923–930; doi: 10.1093/bioinformatics/btt656 24227677

[55] Wang L, Wang S, Li W. RSeQC: quality control of RNA-Seq experiments. Bioinformatics 2012;28(16):2184–2185; doi: 10.1093/bioinformatics/bts356 22743226

[56] Anders S, Pyl PT, Huber W. HTSeq–a Python framework to work with high-throughput sequencing data. Bioinformatics 2015;31(2):166–169; doi: 10.1093/bioinformatics/btu638 25260700 PMC4287950

[57] Wagner GP, Kin K, Lynch VJ. Measurement of mRNA abundance using RNA-Seq data: RPKM measure is inconsistent among samples. Theor Biosci 2012;131(4):281–285; doi: 10.1007/s12064-012-0162-3 22872506

[58] Lawrence M, Huber W, Pages H, et al. Software for computing and annotating genomic ranges. PLoS Comput Biol 2013;9(8):e1003118; doi: 10.1371/journal.pcbi.1003118 23950696 PMC3738458

[59] Haas BJ, Papanicolaou A, Yassour M, et al. De novo transcript sequence reconstruction from RNA-Seq using the Trinity platform for reference generation and analysis. Nat Protoc 2013;8(8):1494–1512; doi: 10.1038/nprot.2013.084 23845962 PMC3875132

[60] NCBI Resource Coordinators. Database resources of the National Center for Biotechnology Information. Nucleic Acids Res 2016;44(D1):D7–D19; doi: 10.1093/nar/gkv1290 26615191 PMC4702911

[61] Love MI, Huber W, Anders S. Moderated estimation of fold change and dispersion for RNA-Seq data with DESeq2. Genome Biol 2014;15(12):550; doi: 10.1186/s13059-014-0550-8 25516281 PMC4302049

[62] Ashburner M, Ball CA, Blake JA, et al. Gene ontology: tool for the unification of biology. The Gene Ontology Consortium. Nat Genet 2000;25(1):25–29; doi: 10.1038/75556 10802651 PMC3037419

[63] Kanehisa M, Goto S. KEGG: kyoto encyclopedia of genes and genomes. Nucleic Acids Res 2000;28(1):27–30; doi: 10.1093/nar/28.1.27 10592173 PMC102409

[64] Yu GC, Wang LG, Han YY, et al. clusterProfiler: an R Package for Comparing Biological Themes Among Gene Clusters. Omics 2012;16(5):284–287; doi: 10.1089/omi.2011.0118 22455463 PMC3339379

[65] Mailman MD, Feolo M, Jin Y, et al. The NCBI dbGaP database of genotypes and phenotypes. Nat Genet 2007;39(10):1181–1186; doi: 10.1038/ng1007-1181 17898773 PMC2031016

[66] Di Giovanni S, Knoblach SM, Brandoli C, et al. Gene profiling in spinal cord injury shows role of cell cycle in neuronal death. Ann Neurol 2003;53(4):454–468. doi: 10.1002/ana.10472 12666113

[67] Befort K, Karchewski L, Lanoue C, et al. Selective up-regulation of the growth arrest DNA damage-inducible gene Gadd45 alpha in sensory and motor neurons after peripheral nerve injury. Eur J Neurosci 2003;18(4):911–922; doi: 10.1046/j.1460-9568.2003.02827.x 12925017

[68] Blesch A, Lu P, Tsukada S, et al. Conditioning lesions before or after spinal cord injury recruit broad genetic mechanisms that sustain axonal regeneration: Superiority to camp-mediated effects. Exp Neurol 2012;235(1):162–173; doi: 10.1016/j.expneurol.2011.12.037 22227059 PMC3334479

[69] Lin CR, Yang CH, Huang CE, et al. GADD45A protects against cell death in dorsal root ganglion neurons following peripheral nerve injury. J Neurosci Res 2011;89(5):689–699; doi: 10.1002/jnr.22589 21337369

[70] Holmes FE, Kerr N, Chen Y-J, et al. Targeted disruption of the orphan receptor Gpr151 does not alter pain-related behaviour despite a strong induction in dorsal root ganglion expression in a model of neuropathic pain. Mol Cell Neurosci 2017;78:35–40; doi: 10.1016/j.mcn.2016.11.010 27913310 PMC5235321

[71] Lee B, Lee J, Jeon Y, et al. Promoting axon regeneration by enhancing the non-coding function of the injury-responsive coding gene Gpr151. bioRxiv 2021; doi: 10.1101/2021.02.19.431965

[72] Robinson M, Perez MCP, Tebar L, et al. FLRT3 is expressed in sensory neurons after peripheral nerve injury and regulates neurite outgrowth. Mol Cell Neurosci 2004;27(2):202–214; doi: 10.1016/j.mcn.2004.06.008 15485775

[73] Tanabe K, Bonilla I, Winkles JA, et al. Fibroblast growth factor-inducible-14 is induced in axotomized neurons and promotes neurite outgrowth. J Neurosci 2003;23(29):9675–9686; doi: 10.1523/jneurosci.23-29-09675.2003 14573547 PMC6740475

[74] Tsuji L, Yamashita T, Kubo T, et al. FLRT3, a cell surface molecule containing LRR repeats and a FNIII domain, promotes neurite outgrowth. Biochem Biophys Res Commun 2004;313(4):1086–1091; doi: 10.1016/j.bbrc.2003.12.047 14706654

[75] Crisponi L, Buers I, Rutsch F. CRLF1 and CLCF1 in Development, health and disease. Int J Mol Sci 2022;23(2):992; doi: 10.3390/ijms23020992 35055176 PMC8780587

[76] Hobson SA, Bacon A, Elliot-Hunt CR, et al. Galanin acts as a trophic factor to the central and peripheral nervous systems. Cellular and Molecular Life Sciences 2008;65(12):1806–1812; doi: 10.1007/s00018-008-8154-7 18500646 PMC11131753

[77] Wynick D, Thompson SW, McMahon SB. The role of galanin as a multi-functional neuropeptide in the nervous system. Curr Opin Pharmacol 2001;1(1):73–77; doi: 10.1016/S1471-4892(01)00006-6 11712539

[78] Jiang BC, Zhang WW, Yang T, et al. Demethylation of G-protein-coupled receptor 151 promoter facilitates the binding of Kruppel-like factor 5 and enhances neuropathic pain after nerve injury in mice. J Neurosci 2018;38(49):10535–10551; doi: 10.1523/JNEUROSCI.0702-18.2018 30373770 PMC6596256

[79] Xia LP, Luo H, Ma Q, et al. GPR151 in nociceptors modulates neuropathic pain via regulating P2X3 function and microglial activation. Brain 2021;144(11):3405–3420; doi: 10.1093/brain/awab245 34244727

[80] Gaudet P, Livstone MS, Lewis SE, et al. Phylogenetic-based propagation of functional annotations within the Gene Ontology consortium. Brief Bioinform 2011;12(5):449–462; doi: 10.1093/bib/bbr042 21873635 PMC3178059

[81] Kumamaru H, Kadoya K, Adler AF, et al. Generation and post-injury integration of human spinal cord neural stem cells. Nat Methods 2018;15(9):723–731; doi: 10.1038/s41592-018-0074-3 30082899

[82] Pereira IM, Marote A, Salgado AJ, et al. Filling the gap: neural stem cells as a promising therapy for spinal cord injury. Pharmaceuticals 2019;12(2):65; doi: 10.3390/ph12020065 31035689 PMC6631328

[83] White N, Sakiyama-Elbert SE. Derivation of specific neural populations from pluripotent cells for understanding and treatment of spinal cord injury. Dev Dyn 2019;248(1):78–87; doi: 10.1002/dvdy.24680 30324766 PMC6640631

[84] Yu C, Xia K, Gong Z, et al. The application of neural stem/progenitor cells for regenerative therapy of spinal cord injury. Curr Stem Cell Res Ther 2019;14(6):495–503; doi: 10.2174/1574888X14666190329095638 30924422

[85] Garcia-Caballero A, Gadotti VM, Stemkowski P, et al. The deubiquitinating enzyme USP5 modulates neuropathic and inflammatory pain by enhancing Cav3.2 channel activity. Neuron 2014;83(5):1144–1158; doi: 10.1016/j.neuron.2014.07.036 25189210

[86] Veldman MB, Bemben MA, Goldman D. Tuba1a gene expression is regulated by KLF6/7 and is necessary for CNS development and regeneration in zebrafish. Mol Cell Neurosci 2010;43(4):370–383; doi: 10.1016/j.mcn.2010.01.004 20123021 PMC2837137

[87] Fu H, Zhao Y, Hu D, et al. Depletion of microglia exacerbates injury and impairs function recovery after spinal cord injury in mice. Cell Death Dis 2020;11(7):528; doi: 10.1038/s41419-020-2733-4 32661227 PMC7359318

[88] Gerber YN, Saint-Martin GP, Bringuier CM, et al. CSF1R inhibition reduces microglia proliferation, promotes tissue preservation and improves motor recovery after spinal cord injury. Front Cell Neurosci 2018;12:368; doi: 10.3389/fncel.2018.00368 30386212 PMC6198221

[89] Okada S, Nakamura M, Katoh H, et al. Conditional ablation of Stat3 or Socs3 discloses a dual role for reactive astrocytes after spinal cord injury. Nat Med 2006;12(7):829–834; doi: 10.1038/nm1425 16783372

[90] Noble BT, Brennan FH, Popovich PG. The spleen as a neuroimmune interface after spinal cord injury. J Neuroimmunol 2018;321:1–11; doi: 10.1016/j.jneuroim.2018.05.007 29957379

[91] Huang JJ, Ma WJ, Yokoyama S. Expression and immunolocalization of Gpnmb, a glioma-associated glycoprotein, in normal and inflamed central nervous systems of adult rats. Brain Behav 2012;2(2):85–96; doi: 10.1002/brb3.39 22574278 PMC3345354

[92] Cavone L, McCann T, Drake LK, et al. A unique macrophage subpopulation signals directly to progenitor cells to promote regenerative neurogenesis in the zebrafish spinal cord. Dev Cell 2021;56(11):1617–1630 e6; doi: 10.1016/j.devcel.2021.04.031 34033756

[93] Goldshmit Y, Matteo R, Sztal T, et al. Blockage of lysophosphatidic acid signaling improves spinal cord injury outcomes. Am J Pathol 2012;181(3):978–992; doi: 10.1016/j.ajpath.2012.06.007 22819724 PMC3432439

[94] Jaerve A, Muller HW. Chemokines in CNS injury and repair. Cell Tissue Res 2012;349(1):229–248; doi: 10.1007/s00441-012-1427-3 22700007

[95] Faden AI, Wu J, Stoica BA, et al. Progressive inflammation-mediated neurodegeneration after traumatic brain or spinal cord injury. Br J Pharmacol 2016;173(4):681–691; doi: 10.1111/bph.13179 25939377 PMC4742301

[96] Chen G, Fang X, Yu M. Regulation of gene expression in rats with spinal cord injury based on microarray data. Mol Med Rep 2015;12(2):2465–2472; doi: 10.3892/mmr.2015.3670 25936407 PMC4464272

[97] Zhang JJ, Xu XX, Liu HT, et al. Astrocytic YAP prevents the demyelination through promoting expression of cholesterol synthesis genes in experimental autoimmune encephalomyelitis. Cell Death Dis 2021;12(10):1–11; doi: 10.1038/s41419-021-04203-8 34611127 PMC8492624

[98] Van Steenwinckel J, Reaux-Le Goazigo A, Pommier B, et al. CCL2 released from neuronal synaptic vesicles in the spinal cord is a major mediator of local inflammation and pain after peripheral nerve injury. J Neurosci 2011;31(15):5865–5875; doi: 10.1523/jneurosci.5986-10.2011 21490228 PMC6622829

[99] Xu X, Zhang J, Li S, et al. Bone Marrow Stromal Cell Antigen 2: is a potential neuroinflammation biomarker of SOD1G93A mouse model of amyotrophic lateral sclerosis in pre-symptomatic stage. Front Neurosci 2021;15; doi: 10.3389/fnins.2021.788730 PMC8858987 35197819

[100] Wahane S, Zhou X, Zhou X, et al. Diversified transcriptional responses of myeloid and glial cells in spinal cord injury shaped by HDAC3 activity. Sci Adv 2021;7(9):eabd8811; doi: 10.1126/sciadv.abd8811 33637528 PMC7909890

[101] Kobayashi M, Konishi H, Sayo A, et al. TREM2/DAP12 Signal elicits proinflammatory response in microglia and exacerbates neuropathic pain. J Neurosci 2016;36(43):11138–11150; doi: 10.1523/JNEUROSCI.1238-16.2016 27798193 PMC6705657

[102] Gaudet AD, Fonken LK, Ayala MT, et al. Spinal cord injury in rats dysregulates diurnal rhythms of fecal output and liver metabolic indicators. J Neurotrauma 2019;36(12):1923–1934; doi: 10.1089/neu.2018.6101 30501584 PMC10027348

[103] Dhar SS, Zhao DY, Lin T, et al. MLL4 is required to maintain broad H3K4me3 peaks and super-enhancers at tumor suppressor genes. Mol Cell 2018;70(5):825; doi: 10.1016/j.molcel.2018.04.028 29861161 PMC6528658

[104] Dickendesher TL, Baldwin KT, Mironova YA, et al. NgR1 and NgR3 are receptors for chondroitin sulfate proteoglycans. Nat Neurosci 2012;15(5):703–712; doi: 10.1038/nn.3070 22406547 PMC3337880

[105] Brockie S, Hong J, Fehlings MG. The role of microglia in modulating neuroinflammation after spinal cord injury. Int J Mol Sci 2021;22(18):9706; doi: 10.3390/ijms22189706 34575871 PMC8470129

[106] Cordaro M, Casili G, Paterniti I, et al. Fumaric acid esters attenuate secondary degeneration after spinal cord injury. J Neurotrauma 2017;34(21):3027–3040, doi: 10.1089/neu.2016.4678 27889959

[107] Abe N, Cavalli V. Nerve injury signaling. Curr Opin Neurobiol 2008;18(3):276–283; doi: 10.1016/j.conb.2008.06.005 18655834 PMC2633416

[108] Bastien D, Lacroix S. Cytokine pathways regulating glial and leukocyte function after spinal cord and peripheral nerve injury. Exp Neurol 2014;258:62–77; doi: 10.1016/j.expneurol.2014.04.006 25017888

[109] Genovese T, Esposito E, Mazzon E, et al. Effects of palmitoylethanolamide on signaling pathways implicated in the development of spinal cord injury. J Pharmacol Exp Ther 2008;326(1):12–23; doi: 10.1124/jpet.108.136903 18367664

[110] Keane RW, Davis AR, Dietrich WD. Inflammatory and apoptotic signaling after spinal cord injury. J Neurotrauma 2006;23(3-4):335–344; doi: 10.1089/neu.2006.23.335 16629620

[111] Yu CG, Yezierski RP. Activation of the ERK1/2 signaling cascade by excitotoxic spinal cord injury. Brain Res Mol Brain Res 2005;138(2):244–255; doi: 10.1016/j.molbrainres.2005.04.013 15922485

[112] Chen KN, Deng SY, Lu HZ, et al. RNA-Seq characterization of spinal cord injury transcriptome in acute/subacute phases: a resource for understanding the pathology at the systems level. Plos One 2013;8(8):e72567; doi: 10.1371/journal.pone.0072567 23951329 PMC3739761

[113] Duran RC, Yan H, Zheng Y, et al. The systematic analysis of coding and long non-coding RNAs in the sub-chronic and chronic stages of spinal cord injury. Sci Rep 2017;7(1):41008; doi: 10.1038/srep41008 28106101 PMC5247719

[114] Jermakowicz WJ, Carballosa-Gautam MM, Vitores AA, et al. Brainstem-evoked transcription of defensive genes after spinal cord injury. Front Cell Neurosci 2019;13:510; doi: 10.3389/fncel.2019.00510 31803022 PMC6877476

[115] Patel M, Anderson J, Lei S, et al. Nkx6.1 enhances neural stem cell activation and attenuates glial scar formation and neuroinflammation in the adult injured spinal cord. Exp Neurol 2021;345:113826;doi: 10.1016/j.expneurol.2021.113826 34343529 PMC9050175

[116] Satzer D, Miller C, Maxon J, et al. T cell deficiency in spinal cord injury: altered locomotor recovery and whole-genome transcriptional analysis. BMC Neurosci 2015;16:74; doi: 10.1186/s12868-015-0212-0 26546062 PMC4635574

[117] Squair JW, Tigchelaar S, Moon KM, et al. Integrated systems analysis reveals conserved gene networks underlying response to spinal cord injury. Elife 2018;7:e39188; doi: 10.7554/eLife.39188 30277459 PMC6173583

[118] Uttam S, Wong C, Amorim IS, et al. Translational profiling of dorsal root ganglia and spinal cord in a mouse model of neuropathic pain. Neurobiol Pain 2018;4:35–44; doi: 10.1016/j.ynpai.2018.04.001 30906902 PMC6428075

[119] Avraham O, Feng R, Ewan EE, et al. Profiling sensory neuron microenvironment after peripheral and central axon injury reveals key pathways for neural repair. Elife 2021;10:e68457, doi: 10.7554/eLife.68457 34586065 PMC8480984

[120] Floriddia EM, Lourenco T, Zhang SP, et al. Distinct oligodendrocyte populations have spatial preference and different responses to spinal cord injury. Nat Comm 2020;11(1):1–15; doi: 10.1038/s41467-020-19453-x PMC7673029 33203872

[121] Hou JX, Bi HR, Ge QT, et al. Heterogeneity analysis of astrocytes following spinal cord injury at single-cell resolution. FASEB J 2022;36(8):e22442; doi: 10.1096/fj.202200463R 35816276

[122] Li Y, He X, Kawaguchi R, et al. Microglia-organized scar-free spinal cord repair in neonatal mice. Nature 2020;587(7835):613–618; doi: 10.1038/s41586-020-2795-6 33029008 PMC7704837

[123] Matson KJE, Russ DE, Kathe C, et al. Single cell atlas of spinal cord injury in mice reveals a pro-regenerative signature in spinocerebellar neurons. Nat Commun 2022;13(1):5628; doi: 10.1038/s41467-022-33184-1 36163250 PMC9513082

[124] Squair JW, Gautier M, Kathe C, et al. Confronting false discoveries in single-cell differential expression. Nat Commun 2021;12(1):5692; doi: 10.1038/s41467-021-25960-2 34584091 PMC8479118

[125] Wang J, Xu L, Lin W, et al. Single-cell transcriptome analysis reveals the immune heterogeneity and the repopulation of microglia by Hif1alpha in mice after spinal cord injury. Cell Death Dis 2022;13(5):432; doi: 10.1038/s41419-022-04864-z 35504882 PMC9065023

