# Supplementary figures and images for: Construction of a Searchable Database for Gene Expression Changes in Spinal Cord Injury Experiments
Source: J Neurotrauma. 2024 May 25;41(9-10):1030–43. doi: 10.1089/neu.2023.0035 (PMC11302316; doi:10.1089/neu.2023.0035)

**Supplemental Figure S1: Injury location in filtered RNA-Seq experiments.**

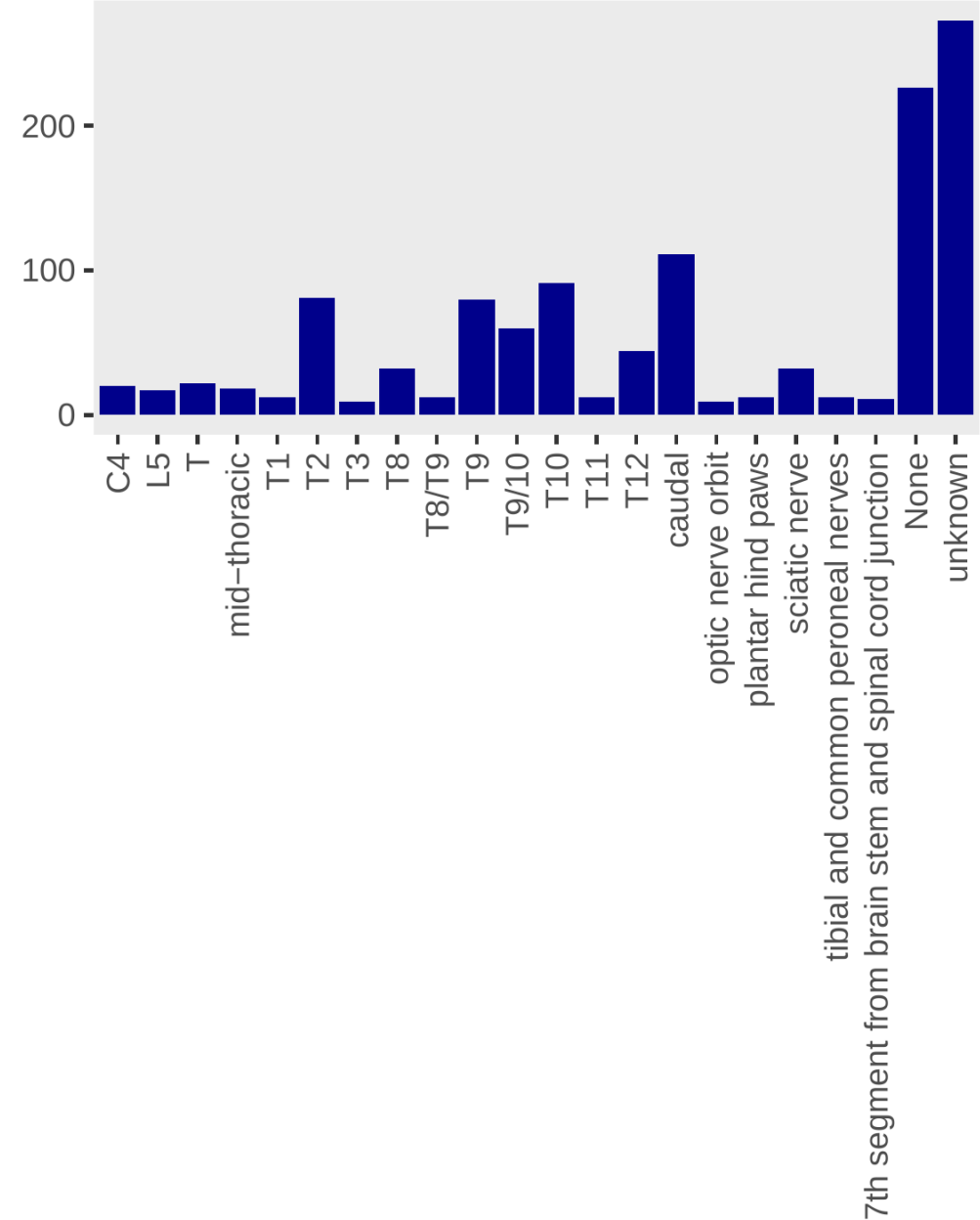

Supplement: Supplementary Figure S1 [file neu.2023.0035_suppl_figures1.pdf]

**Supplemental Figure S2: Time since injury for collection date in filtered RNA-Seq experiments.**

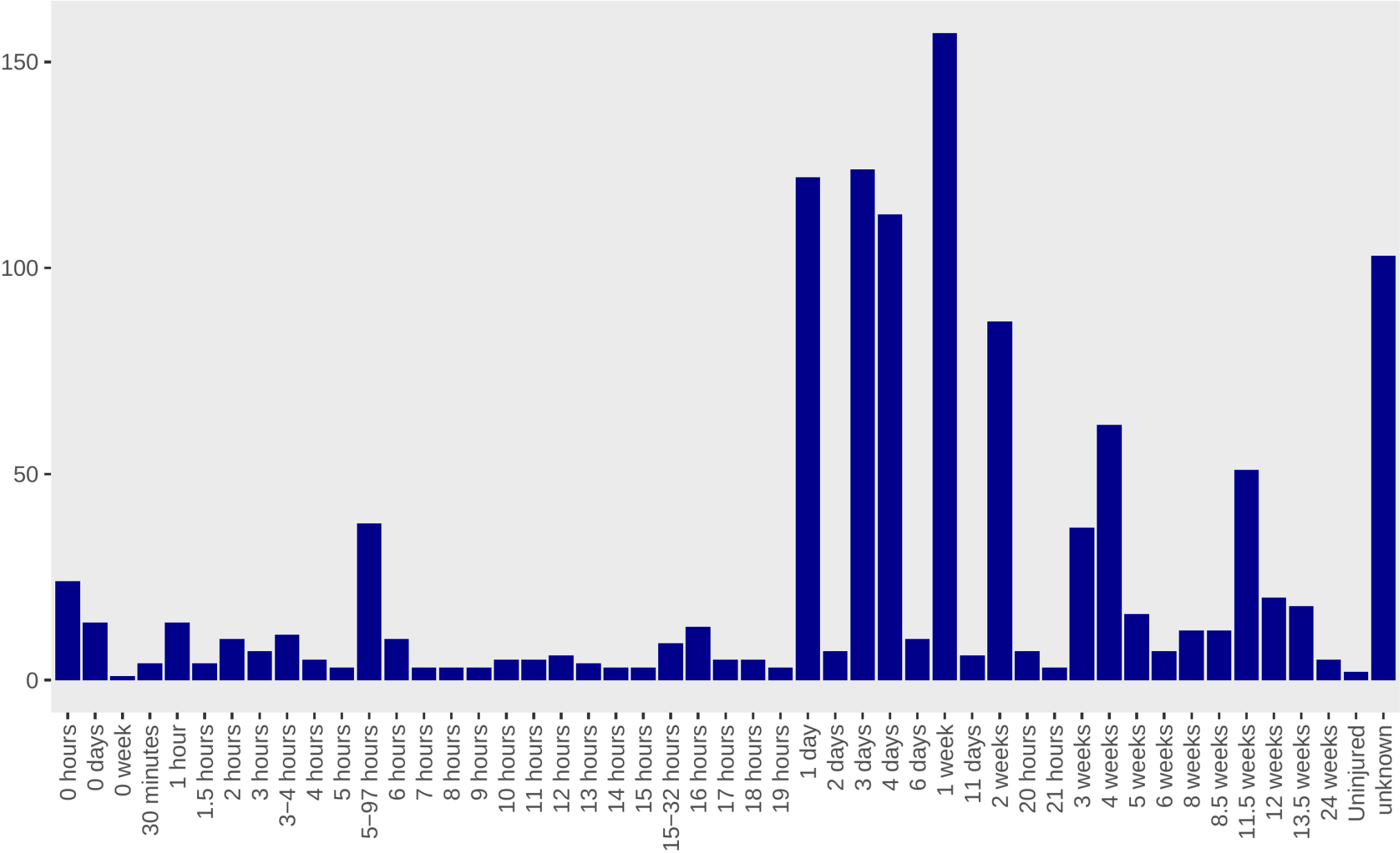

Supplement: Supplementary Figure S2 [file neu.2023.0035_suppl_figures2.pdf]

Supplemental Figure S3: Injury type for filtered RNA-Seq experiments.

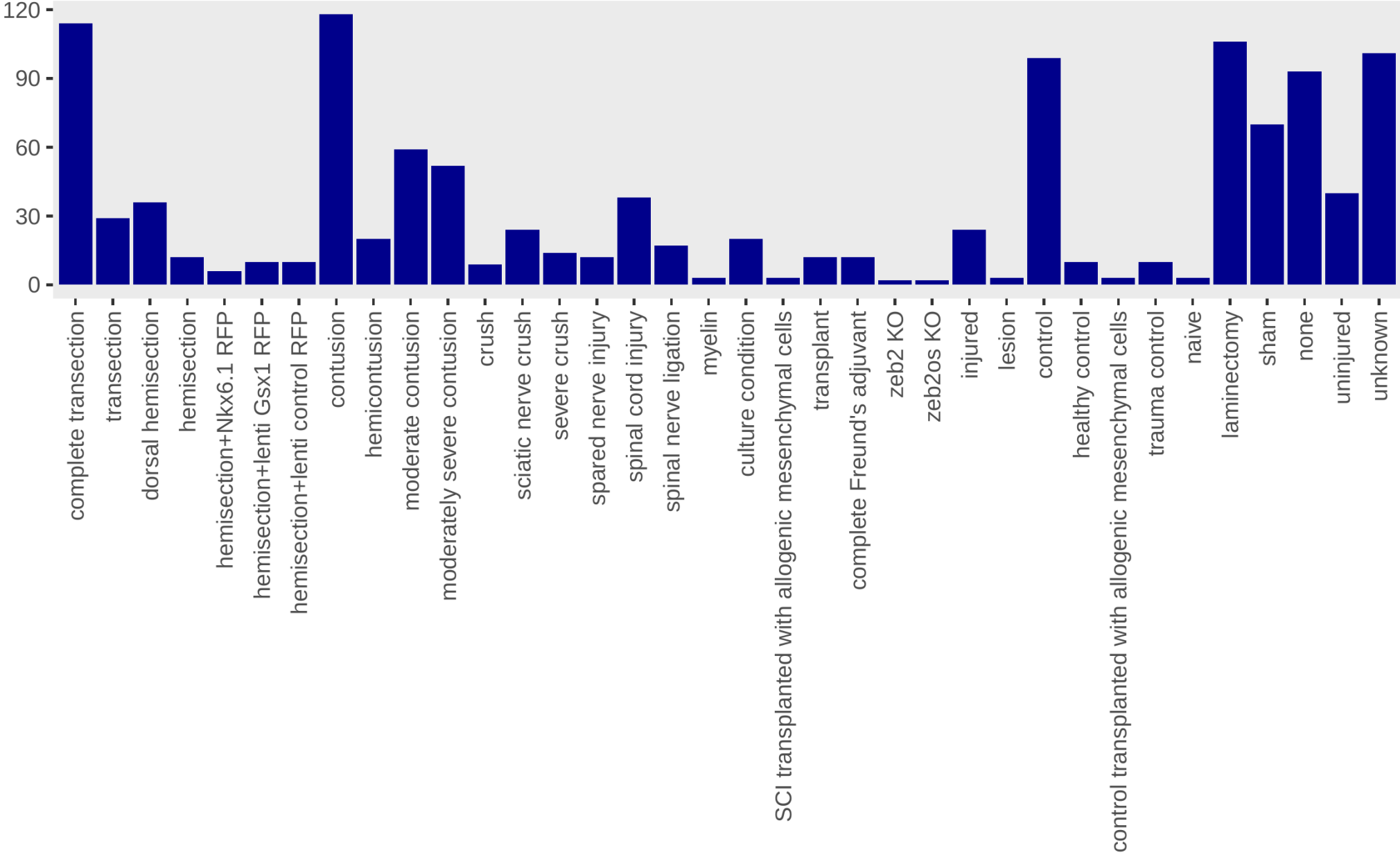

Supplement: Supplementary Figure S3 [file neu.2023.0035_suppl_figures3.pdf]

Supplemental Figure S8: Web results for differentially expressed genes.

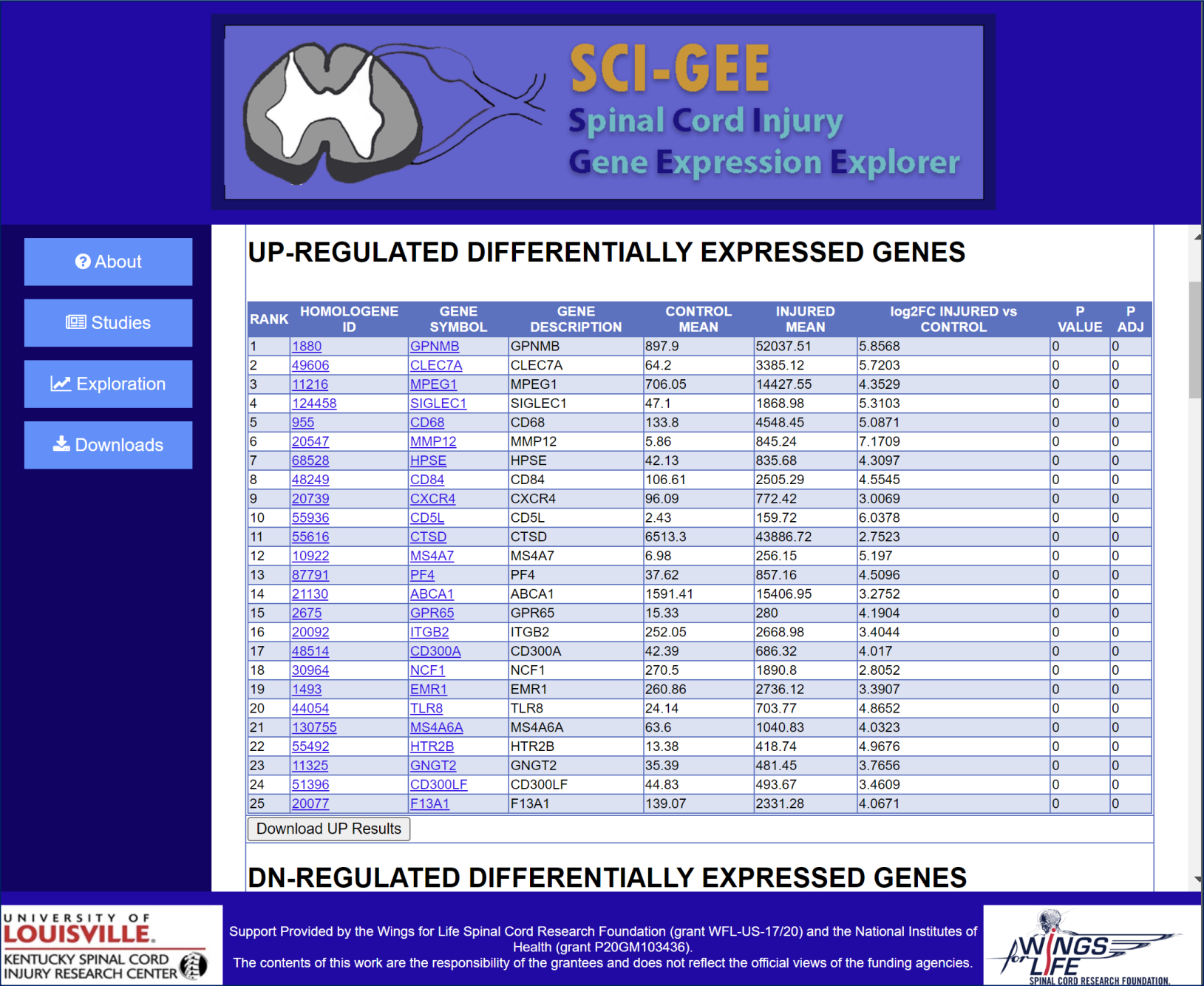

Supplement: Supplementary Figure S8 [file neu.2023.0035_suppl_figures8.pdf]
