# Supplementary material for: Construction of a Searchable Database for Gene Expression Changes in Spinal Cord Injury Experiments
Source: J Neurotrauma. 2024 May 25;41(9-10):1030–43. doi: 10.1089/neu.2023.0035 (PMC11302316; doi:10.1089/neu.2023.0035)
Supplement: Supplementary Figure S5 [file neu.2023.0035_suppl_figures5.pdf]

Supplemental Figure S5: Summary of studies included in SCI-GEE and the SQLite database.

SCI-GEE
Spinal Cord Injury
Gene Expression Explorer

About
Studies
Exploration
Downloads

Sequence Read Archive Studies in SCI-GEE

SRA ID	ORG	STUDY TITLE	NUM SAMPLES	SUBMIT DATE	PUBMED
SRP255811	Am	Preclinical molecular signatures of spinal cord functional restoration: optimizing the metamorphic axolotl (Ambystoma mexicanum) model in regenerative medicine	4	2020-04-10	
SRP255836	Am	Identification of the molecular signatures and functional restoration of the spinal cord of metamorphic axolotl	4	2020-04-10	
SRP334274	Dr	Next Generation Sequencing of zebrafish intraspinal serotonergic neurons in the injury segment and distal segments after spinal cord injury	11	2021-11-09	34876587
SRP259365	Hs	Generation of induced motor neurons (iMNs) from human fibroblasts facilitates locomotor recovery after spinal cord injury	7	2020-06-29	32571478
SRP265127	Hs	Blood RNA biomarkers for spinal cord injury	58	2021-01-22	33512429
SRP220569	Md	Identification of regenerative processes in neonatal spinal cord injury in the opossum (Monodelphis domestica)	42	2020-10-05	
DRP003667	Mm	Genome-wide expression analysis of reactive astrocytes in the injured spinal cord at 7 days after spinal cord injury, host astrocytes in the naive spianl cord, and transplanted astrocytes in the naive spianl cord at 7 days after being transplanted	9	2017-06-07	
DRP003669	Mm	Genome-wide expression analysis in the naive spinal cord and the injured spinal cord at 14 day after spinal cord injury	2	2017-06-07	
SRP019916	Mm	RNA-Seq characterization of spinal cord injury transcriptome in acute/subacute phases: a resource for understanding the pathology at the systems level	8	2013-08-28	23951329
SRP049253	Mm	Spinal cord injury (RNA sequencing data)	44	2014-12-03	25385836
SRP067494	Mm	In vivo analysis of astrocyte ribosome-associated mRNA after traumatic spinal cord injury	22	2016-03-30	
SRP079387	Mm	Macrophage transcriptional profile identifies lipid catabolic pathways that can be therapeutically targeted after spinal cord injury	6	2017-01-30	28130359
SRP094587	Mm	Characterization of meningeal type 2 innate lymphocytes and their response to CNS injury	54	2016-12-14	27994070
SRP097644	Mm	In vivo analysis of injury sites presenting full or attenuated pericyte-derived scarring after spinal cord injury (SCI)	12	2018-02-27	29502968
SRP101665	Mm	Time-course analysis of astrocyte-specific RNA-seq in two severities of spinal cord injury	12	2017-03-12	27716282
SRP101667	Mm	Time-course analysis of microglia-specific RNA-seq in two severities of spinal cord injury	20	2017-03-27	28420963
SRP133622	Mm	Mouse transcriptomics reveals extracellular matrix organization as a major pathway involved in inflammatory and neuropathic pain	36	2019-04-04	30763288
SRP142367	Mm	Microglia and macrophages promote corralling, wound compaction and recovery in spinal cord injury via Plexin-B2	12	2019-12-26	32112058
SRP173586	Mm	Translational profiling of dorsal root ganglia and spinal cord in a mouse model of neuropathic pain	32	2018-12-18	30906902
SRP179750	Mm	Cellular response of mesenchymal stem cells transplanted into spinal cord injury	44	2019-04-23	30944028
SRP201114	Mm	Transcriptional changes after spinal cord injury: recruitment of afferents distal to the site of injury	108	2019-11-28	
SRP226573	Mm	Syngeneic, in contrast to allogeneic, mesenchymal stem cells have superior therapeutic potential following spinal cord injury	12	2019-10-29	
SRP259320	Mm	Ascending dorsal column sensory neurons respond to spinal cord injury and downregulate genes related to lipid metabolism	74	2021-01-19	33431991
SRP269775	Mm	Systematic analysis of purified astrocytes after spinal cord injury unveils lncRNA Zeb2os as a novel molecular target for astrogliosis [RNA-Seq]	25	2021-02-09	33535036
[illegible]	[illegible]	[illegible]	[illegible]	[illegible]	33895323

UNIVERSITY OF LOUISVILLE. KENTUCKY SPINAL CORD INJURY RESEARCH CENTER

Support Provided by the Wings for Life Spinal Cord Research Foundation (grant WFL-US-17/20) and the National Institutes of Health (grant P20GM103436).
The contents of this work are the responsibility of the grantees and does not reflec t the official views of the funding agencies.

WINGS for LIFE SPINAL CORD RESEARCH FOUNDATION
