# Supplementary material for: Construction of a Searchable Database for Gene Expression Changes in Spinal Cord Injury Experiments
Source: J Neurotrauma. 2024 May 25;41(9-10):1030–43. doi: 10.1089/neu.2023.0035 (PMC11302316; doi:10.1089/neu.2023.0035)

Supplemental Figure S7: **Sample selection page**. Users are allowed to check individual samples to incorporate in a pairwise comparison, label the individual sample groups, and identify the sample group to use as the baseline comparison.

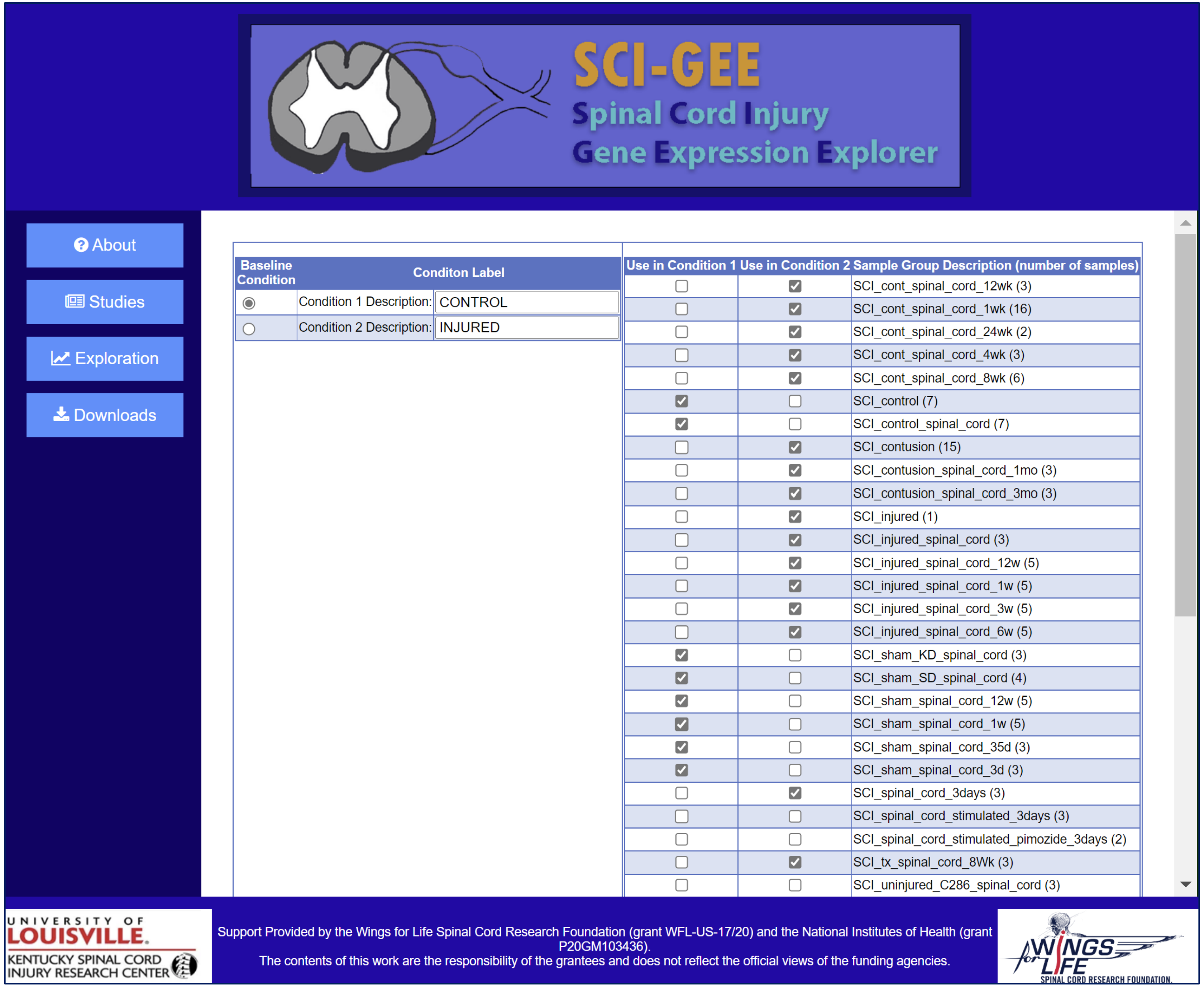

Supplement: Supplementary Figure S7 [file neu.2023.0035_suppl_figures7.pdf]
