# Supplementary material for: Construction of a Searchable Database for Gene Expression Changes in Spinal Cord Injury Experiments
Source: J Neurotrauma. 2024 May 25;41(9-10):1030–43. doi: 10.1089/neu.2023.0035 (PMC11302316; doi:10.1089/neu.2023.0035)

Supplemental Figure S9: **Example heatmap of top differentially expressed genes.** Samples are shown across the columns, and individual genes are shown across the rows.

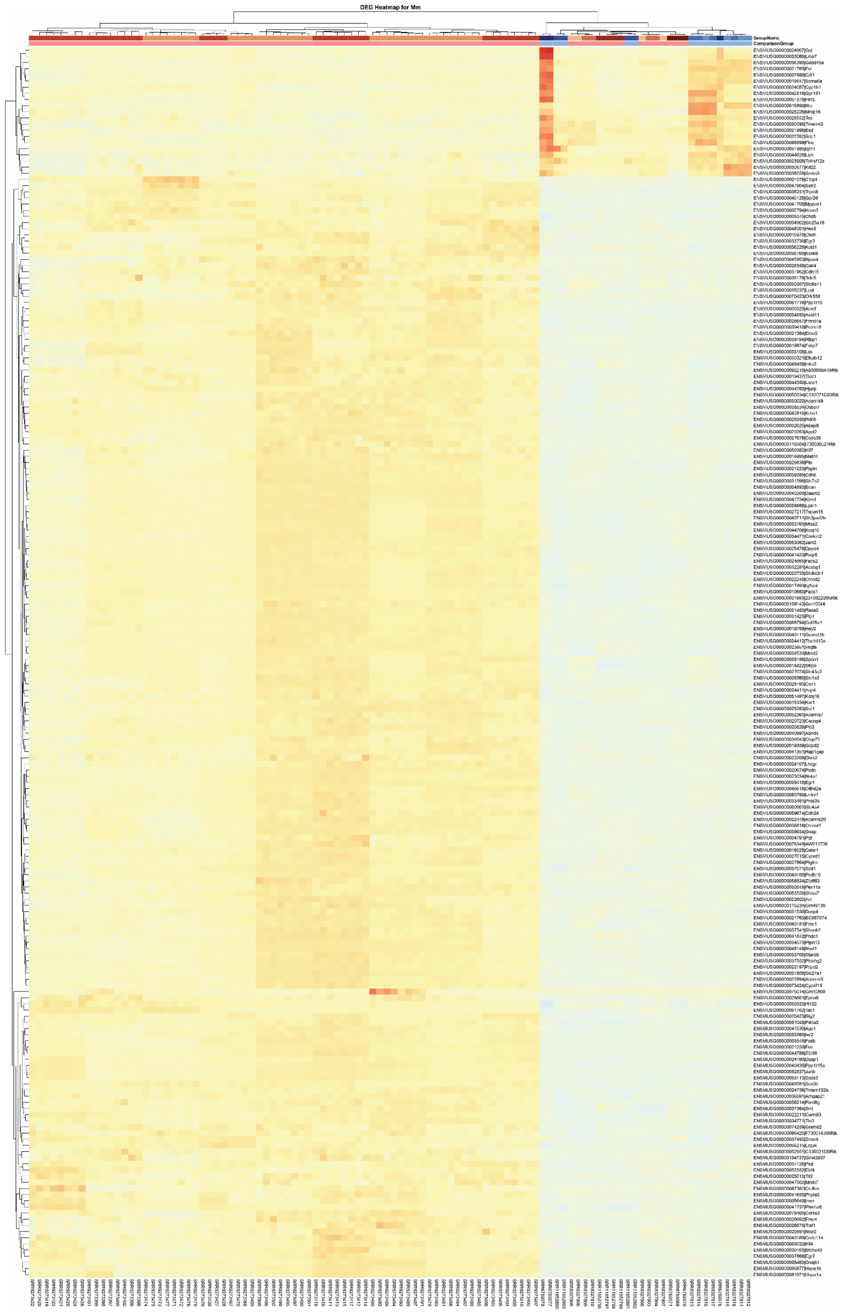

Supplement: Supplementary Figure S9 [file neu.2023.0035_suppl_figures9.pdf]
