# Supplementary material for: Construction of a Searchable Database for Gene Expression Changes in Spinal Cord Injury Experiments
Source: J Neurotrauma. 2024 May 25;41(9-10):1030–43. doi: 10.1089/neu.2023.0035 (PMC11302316; doi:10.1089/neu.2023.0035)

Supplemental Figure S10: Heatmaps for top differentially expressed genes in DRG injury vs. control comparison for (A) meta-analysis of mouse and rat homologs; (B) mouse samples; and (C) rat samples.

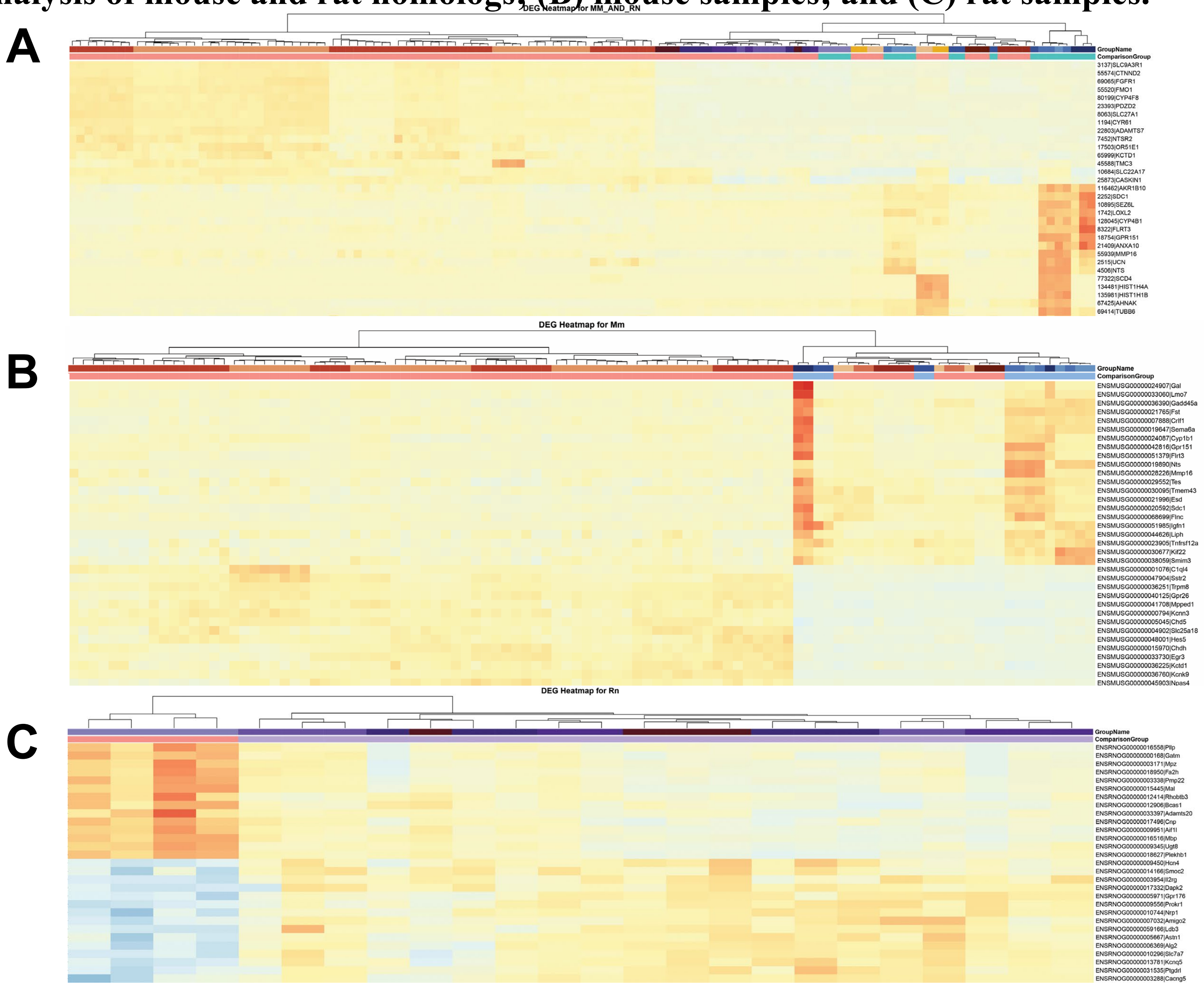

Supplement: Supplementary Figure S10 [file neu.2023.0035_suppl_figures10.pdf]
