# Supplementary material for: Construction of a Searchable Database for Gene Expression Changes in Spinal Cord Injury Experiments
Source: J Neurotrauma. 2024 May 25;41(9-10):1030–43. doi: 10.1089/neu.2023.0035 (PMC11302316; doi:10.1089/neu.2023.0035)
Supplement: Supplementary Table S1 [file neu.2023.0035_suppl_tables1.pdf]

Supplemental Table S1: List of high-throughput sequencing studies in SRA related to "spinal cord injury" or SCI.

SRA Accession	Assay	Organism	Title	# of Samples	Release Date
SRP255811	RNA-Seq	Am	Preclinical molecular signatures of spinal cord functional restoration: optimizing the metamorphic axolotl (Ambystoma mexicanum) model in regenerative medicine	4	2020-04-10
SRP255836	RNA-Seq	Am	Identification of the molecular signatures and functional restoration of the spinal cord of metamorphic axolotl	4	2020-04-10
SRP269070	RIP-Seq	Dr	Changes of m6A RNA methylation following spinal cord injury	4	2020-10-23
SRP334274	RNA-Seq	Dr	Next Generation Sequencing of zebrafish intraspinal serotonergic neurons in the injury segment and distal segments after spinal cord injury	11	2021-11-09
SRP065292	WXS	Epsilonproteobacteria	bacterial symbionts associated with Shinkaia Crosnieri from Iheya North raw sequence reads	1	2016-01-06
SRP198857	OTHER	feces metagenome	Gut microbiota dysbiosis in spinal cord injury	60	2020-06-14
SRP078936	AMPLICON	gut metagenome	feces metagenome Raw sequence reads	6	2016-10-04
SRP148843	AMPLICON	gut metagenome	spinal cord injury and gut microbiota	23	2019-06-24
SRP158549	AMPLICON	gut metagenome	gut microbioal community diversity of spinal cord injury	93	2019-08-31
SRP287631	AMPLICON	gut metagenome	Profile of the Gut Microbiota in Traumatic Thoracic Spinal Cord Injury	69	2020-10-19
SRP316106	AMPLICON	gut metagenome	Gut microbiota in patients during the acute phase after spinal cord injury: a multicenter study in Italian spinal units	100	2021-04-24
SRP351907	AMPLICON	gut metagenome	Effect of moxibustion on intestinal bacteria in rats with spinal cord injury	36	2021-12-21
SRP362110	AMPLICON	gut metagenome	Mus musculus feces 16S rRNA Raw sequence reads	20	2022-04-02
SRP386175	AMPLICON	Hs	The gut microbiota of patients with Spinal Cord Injury	21	2022-07-15
SRP321576	ATAC-seq	Hs	Cell-specific chromatin landscape in human coronary artery resolves regulatory mechanisms of disease risk	2	2021-12-17
SRP259365	RNA-Seq	Hs	Generation of induced motor neurons (iMNs) from human fibroblasts facilitates locomotor recovery after spinal cord injury	7	2020-06-29
SRP265127	RNA-Seq	Hs	Blood RNA biomarkers for spinal cord injury	58	2021-01-22
SRP316185	RNA-Seq Transplanted	Hs	Mouse spinal cords transplanted with human iPS cell-derived neural cells with and without chemogenetic stimulations on 14-days and 42-days after spinal cord injury	19	2021-11-24
SRP211139	CLONE	La	Sequencing LOXODONTA AFRICANA	1	2020-01-01
SRP220569	RNA-Seq	Md	Identification of regenerative processes in neonatal spinal cord injury in the opossum (Monodelphis domestica)	42	2020-10-05
SRP268495	ChIP-Seq	Mm	Systematic analysis of purified astrocytes after spinal cord injury unveils lncRNA Zeb2os as a novel molecular target for astrogliosis [ChIP-Seq]	4	2021-02-09
SRP092566	miRNA-Seq	Mm	MicroRNA expression profiling in a mouse spinal cord injury model [P15 and P90, 12h and 3d]	24	2018-02-12
SRP093778	miRNA-Seq	Mm	MicroRNA expression profiling in a mouse spinal cord injury model [P90, 7d]	6	2018-02-12
SRP283432	miRNA-Seq	Mm	Identification of miRNA expression profiles following traumatic spinal cord injury by high-throughput sequence analysis [miRNA]	6	2021-10-28
SRP283435	ncRNA-Seq	Mm	Identification of noncoding RNA expression profiles following traumatic spinal cord injury by high-throughput sequence analysis	5	2021-10-28
SRP365039	ncRNA-Seq	Mm	Analysis of long noncoding RNAs expression profiles explores mechanisms of hyperbaric oxygen treatment promoting spinal cord injury recovery	9	2022-03-24
SRP357545	OTHER	Mm	Spatiotemporal dynamics of molecular expression pattern and intercellular interactions in glial scar responding to spinal cord injury	8	2022-02-01
SRP134115	RAM-Seq	Mm	In vivo analysis of spinal cord astrocyte and non-astrocyte gene expression after traumatic spinal cord injury, with or without intraspinal treatment with hydrogel depot containing FGF+EGF	23	2018-09-20

SRA Accession	Assay	Organism	Title	# of Samples	Release Date
DRP003667	RNA-Seq	Mm	Genome-wide expression analysis of reactive astrocytes in the injured spinal cord at 7 days after spinal cord injury, host astrocytes in the naive spianl cord, and transplanted astrocytes in the naive spianl cord at 7 days after being transplanted	9	2017-06-07
DRP003669	RNA-Seq	Mm	Genome-wide expression analysis in the naive spinal cord and the injured spinal cord at 14 day after spinal cord injury	2	2017-06-07
SRP019916	RNA-Seq	Mm	RNA-Seq characterization of spinal cord injury transcriptome in acute/subacute phases: a resource for understanding the pathology at the systems level	8	2013-08-28
SRP049253	RNA-Seq	Mm	Spinal cord injury (RNA sequencing data)	44	2014-12-03
SRP067494	RNA-Seq	Mm	In vivo analysis of astrocyte ribosome-associated mRNA after traumatic spinal cord injury	22	2016-03-30
SRP079387	RNA-Seq	Mm	Macrophage transcriptional profile identifies lipid catabolic pathways that can be therapeutically targeted after spinal cord injury	6	2017-01-30
SRP094587	RNA-Seq	Mm	Characterization of meningeal type 2 innate lymphocytes and their response to CNS injury	54	2016-12-14
SRP097644	RNA-Seq	Mm	In vivo analysis of injury sites presenting full or attenuated pericyte-derived scarring after spinal cord injury (SCI)	12	2018-02-27
SRP101665	RNA-Seq	Mm	Time-course analysis of astrocyte-specific RNA-seq in two severities of spinal cord injury	12	2017-03-12
SRP101667	RNA-Seq	Mm	Time-course analysis of microglia-specific RNA-seq in two severities of spinal cord injury	20	2017-03-27
SRP133622	RNA-Seq	Mm	Mouse transcriptomics reveals extracellular matrix organization as a major pathway involved in inflammatory and neuropathic pain	36	2019-04-04
SRP142367	RNA-Seq	Mm	Microglia and macrophages promote corralling, wound compaction and recovery in spinal cord injury via Plexin-B2	12	2019-12-26
SRP173586	RNA-Seq	Mm	Translational profiling of dorsal root ganglia and spinal cord in a mouse model of neuropathic pain	32	2018-12-18
SRP179750	RNA-Seq	Mm	Cellular response of mesenchymal stem cells transplanted into spinal cord injury	44	2019-04-23
SRP201114	RNA-Seq	Mm	Transcriptional changes after spinal cord injury: recruitment of afferents distal to the site of injury	108	2019-11-28
SRP226573	RNA-Seq	Mm	Syngeneic, in contrast to allogeneic, mesenchymal stem cells have superior therapeutic potential following spinal cord injury	12	2019-10-29
SRP259320	RNA-Seq	Mm	Ascending dorsal column sensory neurons respond to spinal cord injury and downregulate genes related to lipid metabolism	74	2021-01-19
SRP269775	RNA-Seq	Mm	Systematic analysis of purified astrocytes after spinal cord injury unveils lncRNA Zeb2os as a novel molecular target for astrogliosis [RNA-Seq]	25	2021-02-09
SRP313384	RNA-Seq	Mm	Gsx1 Promotes Locomotor Functional Recovery After Spinal Cord Injury	32	2021-05-01
SRP325651	RNA-Seq	Mm	Next Generation Sequencing Facilitates Quantitative Analysis of Wild and spinal cord injury mice	6	2021-06-27
DRP008471	scRNA-Seq	Mm	Single-nucleus RNA sequencing of neonatal and adult mice after spinal cord injury	8	2022-04-23
SRP188838	scRNA-Seq	Mm	Distinct oligodendrocyte populations have spatial preference and different responses to spinal cord injury	5	2020-09-15
SRP239303	scRNA-Seq	Mm	Permanently Re-programmed Microglia in Spinal Cord Injury Contribute to Functional Recovery	3687	2021-12-25
SRP262355	scRNA-Seq	Mm	Scar-free healing and axon regeneration after spinal cord injury in neonatal mice is orchestrated by microglia	10	2020-10-07
SRP269804	scRNA-Seq	Mm	Diversified transcriptional responses of myeloid and glial cells in spinal cord injury shaped by HDAC3 activity	8	2021-01-28
SRP286048	scRNA-Seq	Mm	scRNAseq analysis of mouse L4 whole dorsal root ganglions following sciatic nerve cush, dorsal root crush and spinal cord injury	14	2021-09-14
SRP287675	scRNA-Seq	Mm	Time-resolved single-cell RNAseq profiling identifies a novel Fabp5-expressing subpopulation of inflammatory myeloid cells in chronic spinal cord injury	100	2020-10-23
SRP302124	scRNA-Seq	Mm	Confronting false discoveries in single-cell differential expression	24	2021-08-06

SRA Accession	Assay	Organism	Title	# of Samples	Release Date
SRP315018	scRNA-Seq	Mm	A Single Cell Atlas of Spared Tissue After Spinal Cord Injury Reveals the Mechanisms of Restricted Repair	144	2022-08-03
SRP334371	scRNA-Seq	Mm	Immunel Landscape of Spinal Cord after Injury in Mice Using Single-Cell RNA-Seq	8	2021-08-31
SRP337638	scRNA-Seq	Mm	The neurons that restore walking after paralysis [snRNA-seq]	24	2022-06-21
SRP346706	scRNA-Seq	Mm	Heterogeneity analysis of astrocytes following spinal cord injury at single-cell resolution	8	2021-11-22
SRP352969	scRNA-Seq	Mm	scRNA-seq to examine the reaction of a subpopulation of ependymal cells, EpA cells, to spinal cord injury	4	2022-01-03
SRP186219	WGS	Pa	Pseudomonas aeruginosa Raw sequence reads	2	2021-06-30
SRP101364	RNA-Seq	Pm	RNA-Seq analysis after spinal cord injury in lamprey reveals distinct transcriptional responses during functional recovery in spinal cord and brain	22	2018-03-04
SRP354855	AMPLICON	Rn	T10 Spinal cord tissue in Spinal cord injury rat with Physical exercise therapy	6	2022-01-13
SRP202543	ncRNA-Seq	Rn	Differential Expression profiles of tRNA-Derived Small RNAs in Rats After Traumatic Spinal Cord Injury	8	2020-02-20
SRP049326	RNA-Seq	Rn	T Cell Deficiency in Spinal Cord Injury: Altered Locomotor Recovery and Whole-Genome Transcriptional Analysis	12	2015-10-26
SRP073355	RNA-Seq	Rn	Transcriptome of Sprague-Dawley rats	15	2017-04-15
SRP096190	RNA-Seq	Rn	RNA-Seq analysis of coding and long non-coding RNAs in the sub-chronic and chronic stages of spinal cord injury	11	2017-01-10
SRP131816	RNA-Seq	Rn	Transcriptional screen in the target region of sprouting hindlimb corticospinal fibers after thoracic spinal cord injury in rats	30	2021-01-31
SRP149309	RNA-Seq	Rn	Integrated systems analysis reveals conserved gene networks underlying response to spinal cord injury	15	2018-10-02
SRP166392	RNA-Seq	Rn	EGFR-ERK blockade upregulates TRIM32 signalling cascade and promotes neurogenesis after spinal cord injury	6	2019-07-01
SRP176640	RNA-Seq	Rn	Activity-induced changes in the liver transcriptome after chronic spinal cord injury	37	2019-04-29
SRP179652	RNA-Seq	Rn	Analysis of possible mechanisms behind functional recovery following neural progenitor cell transplantation into spinal cord injury	28	2019-04-25
SRP181953	RNA-Seq	Rn	Transcriptome of dorsal root ganglia caudal to a spinal cord injury with modulated behavioral activity	24	2019-04-29
SRP192162	RNA-Seq	Rn	Transcriptional changes in soleus muscle for rats exposed to different activities after contusion injury to the spinal cord and transcriptional changes in soleus muscle with complete spinal cord transection injury	20	2020-04-10
SRP202013	RNA-Seq	Rn	Brainstem control of transcription after spinal cord injury (SCI)	17	2019-11-07
SRP213314	RNA-Seq	Rn	Transcriptomic analysis of knockdown of a-synuclein after T3 spinal cord injury in rats	9	2019-09-24
SRP216808	RNA-Seq	Rn	Novel drug-like RAR-Beta agonist induces BRCA1 to prevent neuropathic pain	22	2019-09-12
SRP224959	RNA-Seq	Rn	Genome wide analysis of thoracic spinal cord at 5 days after T9 hemisection injury	4	2020-05-11
SRP273616	RNA-Seq	Rn	Ketogenic diet-mediated steroid metabolism reprogramming improves the immune microenvironment and myelin growth of spinal cord injury rats through gene analysis and co-expression network analysis	15	2021-03-10
SRP275629	RNA-Seq	Rn	Transcriptome of Subcortical White Matter and Spinal Cord After Spinal Injury and Cortical Stimulation	20	2021-02-16
SRP279076	RNA-Seq	Rn	Circular RNAs expression profiles and potential key molecules incompletely transected spinal cord injury	6	2020-08-28
SRP311591	RNA-Seq	Rn	sequencing for spinal cord injury	6	2021-07-19
SRP303498	scRNA-Seq	Rn	Rationally designed, self-assembly, multifunctional hydrogel depot repairs severe spinal cord injury	12	2022-01-02
SRP215000	miRNA-Seq	Rodent	serum exosomal microRNAs in Rats 7 days after spinal cord injury	6	2019-08-27
SRP082514	miRNA-Seq	Ss	miRNA-Seq of miRNA in Sus scrofa serum after spinal cord injury	64	2016-08-29

SRA Accession	Assay	Organism	Title	# of Samples	Release Date
SRP082501	RNA-Seq	Ts	Injured spinal cord from Trachemys scripta elegans 4 dpl N3	6	2017-02-17
SRP273806	miRNA-Seq	Xl	Xenopus laevis small RNA transcriptome after spinal cord injury	12	2020-12-31
DRP006873	RNA-Seq	Xl	RNA-seq analysis after spinal cord injury in Xenopus laevis	12	2021-01-20
SRP222957	RNA-Seq	Xl	Comparative Gene Expression Profiling between Xenopus Optic Nerve and Spinal Cord Injury to Identify Genes Involved in Successful Regeneration of Vertebrate CNS Axons	51	2020-08-09
SRP300206	RNA-Seq	Xl	Cellular response to spinal cord injury in regenerative and non-regenerative stages in Xenopus laevis	2	2021-01-06
SRP302901	RNA-Seq	Xl	High expression profiling analysis of the early response to spinal cord injury identified a key role for mTORC1 signaling	111	2021-11-11
SRP272355	Targeted-Capture	metagenome	Phylogenomics of Neotropical Lecythidaceae	1	2021-08-17
