# Supplementary material for: Construction of a Searchable Database for Gene Expression Changes in Spinal Cord Injury Experiments
Source: J Neurotrauma. 2024 May 25;41(9-10):1030–43. doi: 10.1089/neu.2023.0035 (PMC11302316; doi:10.1089/neu.2023.0035)
Supplement: Supplementary Table S2 [file neu.2023.0035_suppl_tables2.pdf]

Supplemental Table S2: Samples utilized for DRG usage study

SAMPLE	GROUP NAME	COMP GROUP
SRR11652796	SCI_control_DRG_1d	CONTROL
SRR11652797	SCI_control_DRG_1d	CONTROL
SRR11652798	SCI_control_DRG_1d	CONTROL
SRR11652799	SCI_control_DRG_1d	CONTROL
SRR11652800	SCI_hemisection_DRG_1d	SCI
SRR11652801	SCI_hemisection_DRG_1d	SCI
SRR11652802	SCI_hemisection_DRG_1d	SCI
SRR11652803	SCI_hemisection_DRG_1d	SCI
SRR6789069	ctrl_dorsal_root_ganglia_0d	CONTROL
SRR6789070	ctrl_dorsal_root_ganglia_0d	CONTROL
SRR6789071	ctrl_dorsal_root_ganglia_0d	CONTROL
SRR6789075	sni_dorsal_root_ganglia_1wk	SCI
SRR6789076	sni_dorsal_root_ganglia_1wk	SCI
SRR6789077	sni_dorsal_root_ganglia_1wk	SCI
SRR8327803	sham_dorsal_root_ganglia_4wk	CONTROL
SRR8327804	sham_dorsal_root_ganglia_4wk	CONTROL
SRR8327805	sham_dorsal_root_ganglia_4wk_footprint	CONTROL
SRR8327806	sham_dorsal_root_ganglia_4wk_footprint	CONTROL
SRR8327807	sham_dorsal_root_ganglia_4wk_footprint	CONTROL
SRR8327808	sham_dorsal_root_ganglia_4wk_footprint	CONTROL
SRR8327809	sham_dorsal_root_ganglia_4wk	CONTROL
SRR8327810	sham_dorsal_root_ganglia_4wk	CONTROL
SRR8327811	SCI_transection_dorsal_root_ganglia_4wk_footprint	SCI
SRR8327812	SCI_transection_dorsal_root_ganglia_4wk_footprint	SCI
SRR8327813	SCI_transection_dorsal_root_ganglia_4wk	SCI
SRR8327814	SCI_transection_dorsal_root_ganglia_4wk	SCI
SRR8327815	SCI_transection_dorsal_root_ganglia_4wk_footprint	SCI
SRR8327816	SCI_transection_dorsal_root_ganglia_4wk_footprint	SCI
SRR8327817	SCI_transection_dorsal_root_ganglia_4wk	SCI
SRR8327818	SCI_transection_dorsal_root_ganglia_4wk	SCI
SRR8485310	SCI_cont_DRG_SWIM_13.5wks	CONTROL
SRR8485311	SCI_cont_DRG_SWIM_13.5wks	CONTROL
SRR8485312	SCI_cont_DRG_SWIM_13.5wks	CONTROL
SRR8485313	SCI_cont_DRG_SWIM_13.5wks	CONTROL
SRR8485314	SCI_cont_DRG_SWIM_13.5wks	CONTROL
SRR8485315	SCI_cont_DRG_SWW_13.5wks	CONTROL
SRR8485316	SCI_cont_DRG_SWW_13.5wks	CONTROL
SRR8485317	SCI_cont_DRG_SWW_13.5wks	CONTROL
SRR8485318	SCI_cont_DRG_SWW_13.5wks	CONTROL
SRR8485319	SCI_cont_DRG_SWW_13.5wks	CONTROL
SRR8485320	SCI_tx_DRG_8.5wks	SCI
SRR8485321	SCI_tx_DRG_8.5wks	SCI
SRR8485322	SCI_tx_DRG_8.5wks	SCI
SRR8485323	SCI_tx_DRG_8.5wks	SCI
SRR8485324	sham_DRG_cont_11.5wks	CONTROL
SRR8485325	sham_DRG_cont_11.5wks	CONTROL
SRR8485326	sham_DRG_cont_11.5wks	CONTROL
SRR8485327	sham_DRG_cont_11.5wks	CONTROL
SRR8485328	SCI_cont_DRG_11.5wks	CONTROL
SRR8485329	SCI_cont_DRG_11.5wks	CONTROL
SRR8485330	SCI_cont_DRG_11.5wks	CONTROL
SRR8485331	SCI_cont_DRG_11.5wks	CONTROL
SRR8485332	SCI_cont_DRG_11.5wks	CONTROL
SRR8485333	SCI_cont_DRG_11.5wks	CONTROL
SRR9273395	naive_dorsal_root_ganglion_4d	CONTROL
SRR9273396	naive_dorsal_root_ganglion_4d	CONTROL
SRR9273397	naive_dorsal_root_ganglion_4d	CONTROL
SRR9273398	naive_dorsal_root_ganglion_4d	CONTROL
SRR9273399	naive_dorsal_root_ganglion_4d	CONTROL
SRR9273400	naive_dorsal_root_ganglion_4d	CONTROL
SRR9273401	naive_dorsal_root_ganglion_4d	CONTROL
SRR9273402	naive_dorsal_root_ganglion_4d	CONTROL
SRR9273403	naive_dorsal_root_ganglion_4d	CONTROL
SRR9273404	naive_dorsal_root_ganglion_4d	CONTROL
SRR9273405	naive_dorsal_root_ganglion_4d	CONTROL
SRR9273406	naive_dorsal_root_ganglion_4d	CONTROL

SRR9273407	naive_dorsal_root_ganglion_4d	CONTROL
SRR9273408	naive_dorsal_root_ganglion_4d	CONTROL
SRR9273409	naive_dorsal_root_ganglion_4d	CONTROL
SRR9273410	naive_dorsal_root_ganglion_4d	CONTROL
SRR9273411	naive_dorsal_root_ganglion_4d	CONTROL
SRR9273412	naive_dorsal_root_ganglion_4d	CONTROL
SRR9273413	naive_dorsal_root_ganglion_4d	CONTROL
SRR9273414	naive_dorsal_root_ganglion_4d	CONTROL
SRR9273415	naive_dorsal_root_ganglion_4d	CONTROL
SRR9273416	naive_dorsal_root_ganglion_4d	CONTROL
SRR9273417	naive_dorsal_root_ganglion_4d	CONTROL
SRR9273418	naive_dorsal_root_ganglion_4d	CONTROL
SRR9273419	naive_dorsal_root_ganglion_4d	CONTROL
SRR9273420	naive_dorsal_root_ganglion_4d	CONTROL
SRR9273421	naive_dorsal_root_ganglion_4d	CONTROL
SRR9273422	naive_dorsal_root_ganglion_4d	CONTROL
SRR9273423	naive_dorsal_root_ganglion_4d	CONTROL
SRR9273424	naive_dorsal_root_ganglion_4d	CONTROL
SRR9273425	naive_dorsal_root_ganglion_4d	CONTROL
SRR9273426	naive_dorsal_root_ganglion_4d	CONTROL
SRR9273427	naive_dorsal_root_ganglion_4d	CONTROL
SRR9273428	naive_dorsal_root_ganglion_4d	CONTROL
SRR9273429	naive_dorsal_root_ganglion_4d	CONTROL
SRR9273430	naive_dorsal_root_ganglion_4d	CONTROL
SRR9273467	sham_dorsal_root_ganglion_4d	CONTROL
SRR9273468	sham_dorsal_root_ganglion_4d	CONTROL
SRR9273469	sham_dorsal_root_ganglion_4d	CONTROL
SRR9273470	sham_dorsal_root_ganglion_4d	CONTROL
SRR9273471	sham_dorsal_root_ganglion_4d	CONTROL
SRR9273472	sham_dorsal_root_ganglion_4d	CONTROL
SRR9273473	sham_dorsal_root_ganglion_4d	CONTROL
SRR9273474	sham_dorsal_root_ganglion_4d	CONTROL
SRR9273475	sham_dorsal_root_ganglion_4d	CONTROL
SRR9273476	sham_dorsal_root_ganglion_4d	CONTROL
SRR9273477	sham_dorsal_root_ganglion_4d	CONTROL
SRR9273478	sham_dorsal_root_ganglion_4d	CONTROL
SRR9273479	sham_dorsal_root_ganglion_4d	CONTROL
SRR9273480	sham_dorsal_root_ganglion_4d	CONTROL
SRR9273481	sham_dorsal_root_ganglion_4d	CONTROL
SRR9273482	sham_dorsal_root_ganglion_4d	CONTROL
SRR9273483	sham_dorsal_root_ganglion_4d	CONTROL
SRR9273484	sham_dorsal_root_ganglion_4d	CONTROL
SRR9273485	sham_dorsal_root_ganglion_4d	CONTROL
SRR9273486	sham_dorsal_root_ganglion_4d	CONTROL
SRR9273487	sham_dorsal_root_ganglion_4d	CONTROL
SRR9273488	sham_dorsal_root_ganglion_4d	CONTROL
SRR9273489	sham_dorsal_root_ganglion_4d	CONTROL
SRR9273490	sham_dorsal_root_ganglion_4d	CONTROL
SRR9273491	sham_dorsal_root_ganglion_4d	CONTROL
SRR9273492	sham_dorsal_root_ganglion_4d	CONTROL
SRR9273493	sham_dorsal_root_ganglion_4d	CONTROL
SRR9273494	sham_dorsal_root_ganglion_4d	CONTROL
SRR9273495	sham_dorsal_root_ganglion_4d	CONTROL
SRR9273496	sham_dorsal_root_ganglion_4d	CONTROL
SRR9273497	sham_dorsal_root_ganglion_4d	CONTROL
SRR9273498	sham_dorsal_root_ganglion_4d	CONTROL
SRR9273499	sham_dorsal_root_ganglion_4d	CONTROL
SRR9273500	sham_dorsal_root_ganglion_4d	CONTROL
SRR9273501	sham_dorsal_root_ganglion_4d	CONTROL
SRR9273502	sham_dorsal_root_ganglion_4d	CONTROL
