# Supplementary material for: Construction of a Searchable Database for Gene Expression Changes in Spinal Cord Injury Experiments
Source: J Neurotrauma. 2024 May 25;41(9-10):1030–43. doi: 10.1089/neu.2023.0035 (PMC11302316; doi:10.1089/neu.2023.0035)
Supplement: Supplementary Table S3 [file neu.2023.0035_suppl_tables3.pdf]

Supplemental Table S3: Up-regulated DRG genes across both mouse and rat studies, ranked by adjusted p-value. P-values and adjusted p-values not shown since they are effectively 0.

RANK	HOMOLOGENE ID	GENE SYMBOL	GENE DESCRIPTION	CONTROL MEAN	SCI MEAN	log2FC
1	1449	GADD45A	GADD45A	105.19	236.09	1.1662
2	18754	GPR151	GPR151	8.54	76.55	3.1631
3	8322	FLRT3	FLRT3	105.57	272.1	1.3659
4	3489	CRLF1	CRLF1	10.17	32.68	1.683
5	7724	GAL	GAL	86.07	545.61	2.6642
6	11532	TMEM43	TMEM43	243.06	359.07	0.5629
7	7324	FST	FST	6.8	52.78	2.9544
8	8451	TNFRSF12A	TNFRSF12A	18.21	49.01	1.428
9	116462	AKR1B10	AKR1B10	11.61	32.69	1.4934
10	32426	SEMA6A	SEMA6A	110.05	294.49	1.4199
11	4670	PROCR	PROCR	11.74	27.41	1.2226
12	135981	HIST1H1B	HIST1H1B	0.29	12.05	5.3488
13	4506	NTS	NTS	16.86	178.63	3.4052
14	7474	PKIB	PKIB	77.44	164.09	1.0833
15	17109	IL17RE	IL17RE	2.25	44.13	4.2916
16	2252	SDC1	SDC1	57.78	206.5	1.8373
17	8256	STEAP1	STEAP1	6.03	13.52	1.1641
18	83924	LMO7	LMO7	59.81	142.55	1.2528
19	52256	CCDC36	CCDC36	0.24	5.43	4.4496
20	128045	CYP4B1	CYP4B1	5.45	21.96	2.0105
21	297	STAR	STAR	4.52	23.69	2.3896
22	37481	FLNC	FLNC	31.91	92.29	1.5321
23	16367	NETO1	NETO1	16.49	54.17	1.7151
24	77322	SCD4	SCD4	4.55	69.49	3.9322
25	134481	HIST1H4A	HIST1H4A	1.34	71.73	5.7393
