# Supplementary material for: Construction of a Searchable Database for Gene Expression Changes in Spinal Cord Injury Experiments
Source: J Neurotrauma. 2024 May 25;41(9-10):1030–43. doi: 10.1089/neu.2023.0035 (PMC11302316; doi:10.1089/neu.2023.0035)
Supplement: Supplementary Table S4 [file neu.2023.0035_suppl_tables4.pdf]

**Supplemental Table S4**: Top 25 down-regulated DRG genes across both mouse and rat studies, ranked by adjusted p-value. P-values and adjusted p-values not shown since they are effectively 0.

| RANK | HOMOLOGENE ID | GENE SYMBOL | GENE DESCRIPTION | CONTROL MEAN | SCI MEAN | log2FC |
|---|---|---|---|---|---|---|
| 1 | 31403 | FOSB | FOSB | 1861.37 | 13.06 | -7.1543 |
| 2 | 3844 | FOS | FOS | 6600 | 69.92 | -6.5604 |
| 3 | 56394 | EGR1 | EGR1 | 4451.45 | 169.25 | -4.7169 |
| 4 | 2558 | ZFP36 | ZFP36 | 1328.01 | 100.89 | -3.7184 |
| 5 | 1194 | CYR61 | CYR61 | 2016.97 | 105.38 | -4.2584 |
| 6 | 1612 | NR4A1 | NR4A1 | 3640.08 | 355.64 | -3.3554 |
| 7 | 2941 | SOCS3 | SOCS3 | 1764.43 | 94.34 | -4.2251 |
| 8 | 56539 | INSRR | INSRR | 226.05 | 62.91 | -1.8451 |
| 9 | 15333 | NPAS4 | NPAS4 | 52.63 | 3.36 | -3.9671 |
| 10 | 7390 | JUNB | JUNB | 1796.55 | 207.2 | -3.1161 |
| 11 | 23433 | TRPM8 | TRPM8 | 1829.05 | 467.67 | -1.9675 |
| 12 | 9056 | ARC | ARC | 331.07 | 44.04 | -2.9101 |
| 13 | 56758 | KCNK9 | KCNK9 | 227.27 | 31.14 | -2.8672 |
| 14 | 10822 | ARHGAP21 | ARHGAP21 | 1094.34 | 666.08 | -0.7163 |
| 15 | 17764 | GPR26 | GPR26 | 637.91 | 104.48 | -2.61 |
| 16 | 37923 | EGR3 | EGR3 | 449.13 | 57.75 | -2.959 |
| 17 | 17712 | LACC1 | LACC1 | 144.43 | 58.63 | -1.3006 |
| 18 | 9044 | ACSBG1 | ACSBG1 | 2773.63 | 1017.52 | -1.4467 |
| 19 | 22803 | ADAMTS7 | ADAMTS7 | 261.32 | 59.68 | -2.1303 |
| 20 | 70926 | MTSS1L | MTSS1L | 1747.59 | 553.26 | -1.6593 |
| 21 | 15012 | MPPED1 | MPPED1 | 105.04 | 37.58 | -1.4829 |
| 22 | 4453 | SPON1 | SPON1 | 588.99 | 256.06 | -1.2017 |
| 23 | 18920 | FHDC1 | FHDC1 | 501.62 | 191.63 | -1.3882 |
| 24 | 74564 | LRRC16B | LRRC16B | 1358.72 | 607.95 | -1.1602 |
| 25 | 31406 | BTG2 | BTG2 | 524.04 | 111.25 | -2.2358 |
