# Supplementary material for: Construction of a Searchable Database for Gene Expression Changes in Spinal Cord Injury Experiments
Source: J Neurotrauma. 2024 May 25;41(9-10):1030–43. doi: 10.1089/neu.2023.0035 (PMC11302316; doi:10.1089/neu.2023.0035)
Supplement: Supplementary Table S5 [file neu.2023.0035_suppl_tables5.pdf]

Supplemental Table S5: Top 25 up-regulated genes for the mouse DRG studies only, ranked by adjusted p-value. P-values and adjusted p-values not shown since they are effectively 0.

RANK	GENE ID	GENE SYMBOL	GENE DESCRIPTION	CONTROL MEAN	SCI MEAN	log2FC
1	ENSMUSG00000036390	Gadd45a	growth arrest and DNA-damage-inducible 45 alpha	68.72	180.31	1.3917
2	ENSMUSG00000021765	Fst	follistatin	5.9	46.92	2.9908
3	ENSMUSG00000042816	Gpr151	G protein-coupled receptor 151	6.95	67.53	3.2795
4	ENSMUSG00000051985	Igfn1	immunoglobulin-like and fibronectin type III domain containing 1	0.83	11.37	3.7645
5	ENSMUSG00000029552	Tes	testis derived transcript	31.41	64.34	1.0343
6	ENSMUSG00000007888	Crlf1	cytokine receptor-like factor 1	5.84	23.78	2.0257
7	ENSMUSG00000051379	Flrt3	fibronectin leucine rich transmembrane protein 3	70.15	214.4	1.6116
8	ENSMUSG00000030095	Tmem43	transmembrane protein 43	164.71	270.13	0.7137
9	ENSMUSG00000024907	Gal	galanin and GMAP prepropeptide	68.66	475.09	2.7905
10	ENSMUSG00000033060	Lmo7	LIM domain only 7	34.34	104.87	1.6105
11	ENSMUSG00000030677	Kif22	kinesin family member 22	8.91	27.33	1.6154
12	ENSMUSG00000019890	Nts	neurotensin	14.66	160.45	3.4516
13	ENSMUSG00000019647	Sema6a	sema domain, transmembrane domain (TM), and cytoplasmic domain, (semaphorin) 6A	83.12	246.06	1.5657
14	ENSMUSG00000021996	Esd	esterase D/formylglutathione hydrolase	189.32	380.1	1.0055
15	ENSMUSG00000028226	Mmp16	matrix metallopeptidase 16	42.45	96.83	1.1896
16	ENSMUSG00000038059	Smim3	small integral membrane protein 3	25.54	55.67	1.124
17	ENSMUSG00000068699	Flnc	filamin C, gamma	18.15	69.84	1.9435
18	ENSMUSG00000023905	Tnfrsf12a	tumor necrosis factor receptor superfamily, member 12a	13.29	39.37	1.5661
19	ENSMUSG00000044626	Liph	lipase, member H	1.14	4.18	1.8718
20	ENSMUSG00000020592	Sdc1	syndecan 1	45.25	177.22	1.9694
21	ENSMUSG00000024087	Cyp1b1	cytochrome P450, family 1, subfamily b, polypeptide 1	144.12	286.98	0.9936
22	ENSMUSG00000025089	Gfra1	glial cell line derived neurotrophic factor family receptor alpha 1	250.19	676.72	1.4354
23	ENSMUSG00000029762	Akr1b8	aldo-keto reductase family 1, member B8	7.73	25.98	1.748
24	ENSMUSG00000029819	Npy	neuropeptide Y	1.07	86.66	6.3283
25	ENSMUSG00000025608	Podxl	podocalyxin-like	45.85	109.93	1.2616
