# Supplementary material for: Construction of a Searchable Database for Gene Expression Changes in Spinal Cord Injury Experiments
Source: J Neurotrauma. 2024 May 25;41(9-10):1030–43. doi: 10.1089/neu.2023.0035 (PMC11302316; doi:10.1089/neu.2023.0035)
Supplement: Supplementary Table S6 [file neu.2023.0035_suppl_tables6.pdf]

Supplemental Table S6: Top 25 down-regulated genes for the mouse DRG studies only, ranked by adjusted p-value. P-values and adjusted p-values not shown since they are effectively 0.

RANK	GENE ID	GENE SYMBOL	GENE DESCRIPTION	CONTROL MEAN	SCI MEAN	log2FC
1	ENSMUSG00000003545	Fosb	FBJ osteosarcoma oncogene B	1666.47	7.4	-7.8147
2	ENSMUSG00000021250	Fos	FBJ osteosarcoma oncogene	5908.33	49.15	-6.9093
3	ENSMUSG00000038418	Egr1	early growth response 1	3978.21	115.1	-5.1111
4	ENSMUSG00000044786	Zfp36	zinc finger protein 36	1190.62	56.55	-4.3959
5	ENSMUSG00000053113	Socs3	suppressor of cytokine signaling 3	1576.49	83.84	-4.2328
6	ENSMUSG00000005640	Insrr	insulin receptor-related receptor	186.9	28.98	-2.6888
7	ENSMUSG00000028195	Ccn1	cellular communication network factor 1	1804.86	78.46	-4.5236
8	ENSMUSG00000036760	Kcnk9	potassium channel, subfamily K, member 9	202.01	26.81	-2.9134
9	ENSMUSG00000052837	Junb	jun B proto-oncogene	1592.12	182.02	-3.1287
10	ENSMUSG00000023034	Nr4a1	nuclear receptor subfamily 4, group A, member 1	3250.94	264.97	-3.6169
11	ENSMUSG00000056824	Zfp663	zinc finger protein 663	125.08	12.93	-3.273
12	ENSMUSG00000090877	Hspa1b	heat shock protein 1B	1529.04	137.59	-3.474
13	ENSMUSG00000040125	Gpr26	G protein-coupled receptor 26	563.76	87.4	-2.6893
14	ENSMUSG00000000794	Kcnn3	potassium intermediate/small conductance calcium-activated channel, subfamily N, member 3	130.36	25.81	-2.3361
15	ENSMUSG00000022602	Arc	activity regulated cytoskeletal-associated protein	293.53	36.75	-2.9974
16	ENSMUSG00000004892	Bcan	brevican	3258.87	818.28	-1.9936
17	ENSMUSG00000073424	Cyp4f15	cytochrome P450, family 4, subfamily f, polypeptide 15	263.31	45.81	-2.5228
18	ENSMUSG00000039194	Rlbp1	retinaldehyde binding protein 1	204.11	46.65	-2.1291
19	ENSMUSG00000025350	Rdh5	retinol dehydrogenase 5	256.56	60.7	-2.0793
20	ENSMUSG00000052951	C130021I20Rik	Riken cDNA C130021I20 gene	69.5	12.83	-2.4365
21	ENSMUSG00000039543	Cfap70	cilia and flagella associated protein 70	60.14	9.31	-2.6905
22	ENSMUSG00000053560	Ier2	immediate early response 2	240.62	45.3	-2.4091
23	ENSMUSG00000032363	Adamts7	a disintegrin-like and metallopeptidase (reprolysin type) with thrombospondin type 1 motif, 7	230.94	36.76	-2.6512
24	ENSMUSG00000040260	Daam2	dishevelled associated activator of morphogenesis 2	2726.21	546.06	-2.3197
25	ENSMUSG00000033730	Egr3	early growth response 3	399.99	46.93	-3.0912
