# Supplementary material for: Construction of a Searchable Database for Gene Expression Changes in Spinal Cord Injury Experiments
Source: J Neurotrauma. 2024 May 25;41(9-10):1030–43. doi: 10.1089/neu.2023.0035 (PMC11302316; doi:10.1089/neu.2023.0035)
Supplement: Supplementary Table S7 [file neu.2023.0035_suppl_tables7.pdf]

Supplemental Table S7: Top 25 up-regulated genes for the rat DRG studies only, ranked by adjusted p-value. P-values and adjusted p-values not shown since they are effectively 0.

RANK	GENE ID	GENE SYMBOL	GENE DESCRIPTION	CONTROL MEAN	SCI MEAN	log2FC
1	ENSRNOG00000016141	Hoxc11	homeobox C11	0.09	19.1	7.693
2	ENSRNOG00000001581	Hoxd10	homeo box D10	3.99	132.67	5.0542
3	ENSRNOG00000051472	Hoxd11	homeobox D11	0.61	31.22	5.6608
4	ENSRNOG00000022707	Pmp2	peripheral myelin protein 2	1030.42	3113.61	1.5953
5	ENSRNOG00000003666	Jchain	joining chain of multimeric IgA and IgM	10.08	51.5	2.3522
6	ENSRNOG00000012008	S100a3	S100 calcium binding protein A3	56.96	118.84	1.0608
7	ENSRNOG00000008012	Abcb4	ATP binding cassette subfamily B member 4	644.13	935.07	0.5377
8	ENSRNOG00000003338	Pmp22	peripheral myelin protein 22	33017.76	53934.61	0.7079
9	ENSRNOG00000009345	Ugt8	UDP glycosyltransferase 8	1782.81	2849.37	0.6764
10	ENSRNOG00000003847	Gid4	GID complex subunit 4	2200.07	2626.67	0.2556
11	ENSRNOG00000002776	Sell	selectin L	2.06	17.34	3.0728
12	ENSRNOG00000005934	Mlip	muscular LMNA-interacting protein	890.43	1592.28	0.8385
13	ENSRNOG00000004179	Nts	neurotensin	5.23	42.84	3.0333
14	ENSRNOG00000016558	Pllp	plasmolipin	688.13	1186.04	0.7853
15	ENSRNOG00000000168	Gatm	glycine amidinotransferase	925.96	1271.87	0.4579
16	ENSRNOG00000016516	Mbp	myelin basic protein	18665.76	31593.38	0.7592
17	ENSRNOG00000008451	Fut8	fucosyltransferase 8	1890.82	2420.99	0.3565
18	ENSRNOG00000002820	LOC24906	RoBo-1	6.38	61.96	3.2789
19	ENSRNOG00000008394	Prg2	proteoglycan 2	61.36	222.99	1.8615
20	ENSRNOG00000018627	Plekhb1	pleckstrin homology domain containing B1	7171.99	10332.36	0.5267
21	ENSRNOG00000009951	Aif1l	allograft inflammatory factor 1-like	535.15	848.01	0.6641
22	ENSRNOG00000019057	Prkcq	protein kinase C, theta	307.28	498.68	0.6985
23	ENSRNOG00000004278	Dlx3	distal-less homeobox 3	26.59	61.76	1.2154
24	ENSRNOG00000039405	Prss29	protease, serine, 29	0.65	12.53	4.2614
25	ENSRNOG00000015445	Mal	mal, T-cell differentiation protein	2758.46	5204.02	0.9157
