# Supplementary material for: Construction of a Searchable Database for Gene Expression Changes in Spinal Cord Injury Experiments
Source: J Neurotrauma. 2024 May 25;41(9-10):1030–43. doi: 10.1089/neu.2023.0035 (PMC11302316; doi:10.1089/neu.2023.0035)
Supplement: Supplementary Table S8 [file neu.2023.0035_suppl_tables8.pdf]

Supplemental Table S8: Top 25 down-regulated genes for the rat DRG studies only, ranked by adjusted p-value. P-values and adjusted p-values not shown since they are effectively 0.

RANK	GENE ID	GENE SYMBOL	GENE DESCRIPTION	CONTROL MEAN	SCI MEAN	log2FC
1	ENSRNOG00000015409	Usp5	ubiquitin specific peptidase 5	9757.09	7394.8	-0.3999
2	ENSRNOG00000060728	Tuba1a	tubulin, alpha 1A	38150.46	31911.5	-0.2576
3	ENSRNOG00000018414	Csf1r	colony stimulating factor 1 receptor	1896.26	1086.25	-0.8038
4	ENSRNOG00000028703	Slc39a6	solute carrier family 39 member 6	2975.94	2318.45	-0.3601
5	ENSRNOG00000014046	Sertm1	serine-rich and transmembrane domain containing 1	1052.77	658.05	-0.6779
6	ENSRNOG00000006756	Maged1	MAGE family member D1	12502.64	10170.96	-0.2977
7	ENSRNOG00000010744	Nrp1	neuropilin 1	2642.44	1832.09	-0.5283
8	ENSRNOG00000018255	Clptm1	CLPTM1 regulator of GABA type A receptor forward trafficking	6927.19	5245.38	-0.4012
9	ENSRNOG00000039544	Kcnd1	potassium voltage-gated channel subfamily D member 1	10636.97	7755.31	-0.4558
10	ENSRNOG00000029735	Pid1	phosphotyrosine interaction domain containing 1	972.15	777.58	-0.3222
11	ENSRNOG00000003769	Tmem163	transmembrane protein 163	983.49	735.75	-0.4186
12	ENSRNOG00000011285	Zdhhc22	zinc finger, DHHC-type containing 22	1108.93	740.99	-0.5816
13	ENSRNOG00000013213	Epha4	Eph receptor A4	253.39	152.36	-0.7338
14	ENSRNOG00000022402	Luzp1	leucine zipper protein 1	4778.54	3703.96	-0.3675
15	ENSRNOG00000002361	Prkg2	protein kinase cGMP-dependent 2	601.07	394.97	-0.6057
16	ENSRNOG00000005190	Nipal2	NIPA-like domain containing 2	836.15	637.65	-0.3909
17	ENSRNOG00000033570	Arhgap8	Rho GTPase activating protein 8	266.04	181.04	-0.5552
18	ENSRNOG00000008912	Aqr	aquarius intron-binding spliceosomal factor	2344.44	1802.45	-0.3792
19	ENSRNOG00000003590	Tom1l2	target of myb1 like 2 membrane trafficking protein	7811.77	6068.07	-0.3644
20	ENSRNOG00000001484	Castor2	cytosolic arginine sensor for mTORC1 subunit 2	222.48	139.08	-0.6777
21	ENSRNOG00000003792	Med14	mediator complex subunit 14	1637.5	1321.21	-0.3096
22	ENSRNOG00000001272	Mcm3ap	minichromosome maintenance complex component 3 associated protein	2540.68	2002.93	-0.3431
23	ENSRNOG00000009039	Trappc12	trafficking protein particle complex 12	2815.95	2318.72	-0.2802
24	ENSRNOG00000018251	Mrc1	mannose receptor, C type 1	910.64	528.47	-0.785
25	ENSRNOG00000003954	Il2rg	interleukin 2 receptor subunit gamma	245.73	147.77	-0.7337
