# Supplementary material for: Construction of a Searchable Database for Gene Expression Changes in Spinal Cord Injury Experiments
Source: J Neurotrauma. 2024 May 25;41(9-10):1030–43. doi: 10.1089/neu.2023.0035 (PMC11302316; doi:10.1089/neu.2023.0035)
Supplement: Supplementary Table S9 [file neu.2023.0035_suppl_tables9.pdf]

Supplemental Table S9: Samples utilized for spinal cord usage study.

SAMPLE	GROUP NAME	COMP GROUP
DRR057041	SCI_control	CONTROL
SRR10251520	SCI_control_spinal_cord	CONTROL
SRR10251521	SCI_control_spinal_cord	CONTROL
SRR10251522	SCI_control_spinal_cord	CONTROL
SRR10251523	SCI_control_spinal_cord	CONTROL
SRR10322203	SCI_injured_spinal_cord	SCI
SRR10322204	SCI_injured_spinal_cord	SCI
SRR10322205	SCI_injured_spinal_cord	SCI
SRR10322206	SCI_control_spinal_cord_MSCs	CONTROL
SRR10322207	SCI_control_spinal_cord_MSCs	CONTROL
SRR10322208	SCI_control_spinal_cord_MSCs	CONTROL
SRR10322209	SCI_injured_spinal_cord_MSCs	SCI
SRR10322210	SCI_injured_spinal_cord_MSCs	SCI
SRR10322211	SCI_injured_spinal_cord_MSCs	SCI
SRR10322212	SCI_control_spinal_cord	CONTROL
SRR10322213	SCI_control_spinal_cord	CONTROL
SRR10322214	SCI_control_spinal_cord	CONTROL
SRR12136431	SCI_contusion_spinal_cord_1mo	SCI
SRR12136432	SCI_contusion_spinal_cord_1mo	SCI
SRR12136433	SCI_contusion_spinal_cord_1mo	SCI
SRR12136434	SCI_contusion_spinal_cord_3mo	SCI
SRR12136435	SCI_contusion_spinal_cord_3mo	SCI
SRR12136436	SCI_contusion_spinal_cord_3mo	SCI
SRR12327118	SCI_sham_SD_spinal_cord	CONTROL
SRR12327119	SCI_sham_KD_spinal_cord	CONTROL
SRR12327120	SCI_injury_KD_spinal_cord	SCI
SRR12327121	SCI_injury_KD_spinal_cord	SCI
SRR12327122	SCI_sham_KD_spinal_cord	CONTROL
SRR12327123	SCI_sham_KD_spinal_cord	CONTROL
SRR12327124	SCI_injury_SD_spinal_cord	SCI
SRR12327125	SCI_injury_SD_spinal_cord	SCI
SRR12327126	SCI_injury_SD_spinal_cord	SCI
SRR12327127	SCI_injury_KD_spinal_cord	SCI
SRR12327128	SCI_injury_KD_spinal_cord	SCI
SRR12327129	SCI_injury_KD_spinal_cord	SCI
SRR12327130	SCI_sham_SD_spinal_cord	CONTROL
SRR12327131	SCI_sham_SD_spinal_cord	CONTROL
SRR12327132	SCI_sham_SD_spinal_cord	CONTROL
SRR12374743	SCI_hemicontusion_spinal_cord_2wk	SCI
SRR12374744	SCI_hemicontusion_spinal_cord_2wk	SCI
SRR12374745	SCI_hemicontusion_spinal_cord_2wk	SCI
SRR12374748	SCI_hemicontusion_electrode_spinal_cord_2wk	SCI
SRR12374749	SCI_hemicontusion_electrode_spinal_cord_2wk	SCI
SRR12374750	SCI_hemicontusion_electrode_spinal_cord_2wk	SCI
SRR12374753	SCI_hemicontusion_electrode_1wk_stimulation_spinal_cord_2wk	SCI
SRR12374754	SCI_hemicontusion_electrode_1wk_stimulation_spinal_cord_2wk	SCI
SRR12374755	SCI_hemicontusion_electrode_1wk_stimulation_spinal_cord_2wk	SCI
SRR12374758	SCI_hemicontusion_electrode_1wk_stimulation_spinal_cord_2wk	SCI
SRR12374759	SCI_hemicontusion_electrode_1wk_stimulation_spinal_cord_2wk	SCI
SRR12374760	SCI_hemicontusion_electrode_1wk_stimulation_spinal_cord_2wk	SCI
SRR12534830	SCI_tx_spinal_cord_8Wk	SCI
SRR12534831	SCI_tx_spinal_cord_8Wk	SCI
SRR12534832	SCI_tx_spinal_cord_8Wk	SCI
SRR12534833	sham_spinal_cord_8Wk	CONTROL
SRR12534834	sham_spinal_cord_8Wk	CONTROL
SRR12534835	sham_spinal_cord_8Wk	CONTROL
SRR14140660	SCI_sham_spinal_cord_3d	CONTROL
SRR14140661	SCI_sham_spinal_cord_3d	CONTROL
SRR14140662	SCI_sham_spinal_cord_3d	CONTROL
SRR14140663	SCI_hemisection_spinal_cord_lenti_control_RFP_3d	SCI
SRR14140664	SCI_hemisection_spinal_cord_lenti_control_RFP_3d	SCI
SRR14140665	SCI_hemisection_spinal_cord_lenti_control_RFP_3d	SCI
SRR14140666	SCI_hemisection_spinal_cord_lenti_Gsx1_RFP_3d	SCI
SRR14140667	SCI_hemisection_spinal_cord_lenti_Gsx1_RFP_3d	SCI

SRR14140668	SCI_hemisection_spinal_cord_lenti_Gsx1_RFP_3d	SCI
SRR14140669	SCI_hemisection_spinal_cord_lenti_Nkx6.1_RFP_3d	SCI
SRR14140670	SCI_hemisection_spinal_cord_lenti_Nkx6.1_RFP_3d	SCI
SRR14140671	SCI_hemisection_spinal_cord_lenti_Nkx6.1_RFP_3d	SCI
SRR14140672	SCI_hemisection_spinal_cord_lenti_control_RFP_14d	SCI
SRR14140673	SCI_hemisection_spinal_cord_lenti_control_RFP_14d	SCI
SRR14140674	SCI_hemisection_spinal_cord_lenti_control_RFP_14d	SCI
SRR14140675	SCI_hemisection_spinal_cord_lenti_Gsx1_RFP_14d	SCI
SRR14140676	SCI_hemisection_spinal_cord_lenti_Gsx1_RFP_14d	SCI
SRR14140677	SCI_hemisection_spinal_cord_lenti_Gsx1_RFP_14d	SCI
SRR14140678	SCI_sham_spinal_cord_35d	CONTROL
SRR14140679	SCI_sham_spinal_cord_35d	CONTROL
SRR14140680	SCI_sham_spinal_cord_35d	CONTROL
SRR14140681	SCI_hemisection_spinal_cord_lenti_control_RFP_35d	SCI
SRR14140682	SCI_hemisection_spinal_cord_lenti_control_RFP_35d	SCI
SRR14140683	SCI_hemisection_spinal_cord_lenti_control_RFP_35d	SCI
SRR14140684	SCI_hemisection_spinal_cord_lenti_control_RFP_35d	SCI
SRR14140685	SCI_hemisection_spinal_cord_lenti_Gsx1_RFP_35d	SCI
SRR14140686	SCI_hemisection_spinal_cord_lenti_Gsx1_RFP_35d	SCI
SRR14140687	SCI_hemisection_spinal_cord_lenti_Gsx1_RFP_35d	SCI
SRR14140688	SCI_hemisection_spinal_cord_lenti_Gsx1_RFP_35d	SCI
SRR14140689	SCI_hemisection_spinal_cord_lenti_Nkx6.1_RFP_35d	SCI
SRR14140690	SCI_hemisection_spinal_cord_lenti_Nkx6.1_RFP_35d	SCI
SRR14140691	SCI_hemisection_spinal_cord_lenti_Nkx6.1_RFP_35d	SCI
SRR14916223	SCI_control	CONTROL
SRR14916224	SCI_control	CONTROL
SRR14916225	SCI_control	CONTROL
SRR1632862	SCI_cont_spinal_cord_1wk	SCI
SRR1632863	SCI_cont_spinal_cord_1wk	SCI
SRR1632864	SCI_cont_spinal_cord_1wk	SCI
SRR1632865	SCI_cont_spinal_cord_8wk	SCI
SRR1632866	SCI_cont_spinal_cord_8wk	SCI
SRR1632867	SCI_cont_spinal_cord_8wk	SCI
SRR1632868	SCI_cont_spinal_cord_1wk	SCI
SRR1632869	SCI_cont_spinal_cord_1wk	SCI
SRR1632870	SCI_cont_spinal_cord_1wk	SCI
SRR1632871	SCI_cont_spinal_cord_8wk	SCI
SRR1632872	SCI_cont_spinal_cord_8wk	SCI
SRR1632873	SCI_cont_spinal_cord_8wk	SCI
SRR3027673	sci_crush_spinal_cord_2wk_WT_HA_IP	SCI
SRR3027674	sci_crush_spinal_cord_2wk_WT_Flowthrough	SCI
SRR3027675	sci_crush_spinal_cord_2wk_WT_HA_IP	SCI
SRR3027676	sci_crush_spinal_cord_2wk_WT_Flowthrough	SCI
SRR3027677	sci_crush_spinal_cord_2wk_WT_HA_IP	SCI
SRR3027678	sci_crush_spinal_cord_2wk_WT_Flowthrough	SCI
SRR3027679	sci_crush_spinal_cord_2wk_WT_HA_IP	SCI
SRR3027680	sci_crush_spinal_cord_2wk_WT_Flowthrough	SCI
SRR3027681	sci_crush_spinal_cord_2wk_STAT3KO_HA_IP	SCI
SRR3027682	sci_crush_spinal_cord_2wk_STAT3KO_Flowthrough	SCI
SRR3027683	sci_crush_spinal_cord_2wk_STAT3KO_HA_IP	SCI
SRR3027684	sci_crush_spinal_cord_2wk_STAT3KO_Flowthrough	SCI
SRR3027685	sci_crush_spinal_cord_2wk_STAT3KO_HA_IP	SCI
SRR3027686	sci_crush_spinal_cord_2wk_STAT3KO_Flowthrough	SCI
SRR3395443	SCI_contusion	SCI
SRR3395904	SCI_contusion	SCI
SRR3395970	SCI_contusion	SCI
SRR3406621	SCI_contusion	SCI
SRR3406624	SCI_contusion	SCI
SRR3406630	SCI_contusion	SCI
SRR3406634	SCI_contusion	SCI
SRR3406637	SCI_contusion	SCI
SRR3406640	SCI_contusion	SCI
SRR3406642	SCI_contusion	SCI
SRR3406644	SCI_contusion	SCI
SRR3406646	SCI_contusion	SCI
SRR3406648	SCI_contusion	SCI
SRR3407213	SCI_contusion	SCI
SRR3407214	SCI_contusion	SCI
SRR3945463	sci_contusion_spinal_cord_3d_IP	SCI
SRR3945464	sci_contusion_spinal_cord_3d_IP	SCI

SRR3945465	sci_contusion_spinal_cord_3d_IP	SCI
SRR3945466	sci_contusion_spinal_cord_7d_IP	SCI
SRR3945467	sci_contusion_spinal_cord_7d_IP	SCI
SRR3945468	sci_contusion_spinal_cord_7d_IP	SCI
SRR5151126	sham_spinal_cord_24Wk	CONTROL
SRR5151127	sham_spinal_cord_24Wk	CONTROL
SRR5151128	sham_spinal_cord_24Wk	CONTROL
SRR5151129	SCI_cont_spinal_cord_4wk	SCI
SRR5151130	SCI_cont_spinal_cord_4wk	SCI
SRR5151131	SCI_cont_spinal_cord_4wk	SCI
SRR5151132	SCI_cont_spinal_cord_12wk	SCI
SRR5151133	SCI_cont_spinal_cord_12wk	SCI
SRR5151134	SCI_cont_spinal_cord_12wk	SCI
SRR5151135	SCI_cont_spinal_cord_24wk	SCI
SRR5151136	SCI_cont_spinal_cord_24wk	SCI
SRR5196315	ctrl_spinal_cord_2wk	CONTROL
SRR5196316	ctrl_spinal_cord_2wk	CONTROL
SRR5196317	ctrl_spinal_cord_2wk	CONTROL
SRR5196318	ctrl_spinal_cord_2wk	CONTROL
SRR5312374	SCI_control_spinal_cord	CONTROL
SRR6655778	SCI_injured_spinal_cord_1w	SCI
SRR6655779	SCI_injured_spinal_cord_1w	SCI
SRR6655780	SCI_injured_spinal_cord_1w	SCI
SRR6655781	SCI_injured_spinal_cord_1w	SCI
SRR6655782	SCI_injured_spinal_cord_1w	SCI
SRR6655783	SCI_injured_spinal_cord_3w	SCI
SRR6655784	SCI_injured_spinal_cord_3w	SCI
SRR6655785	SCI_injured_spinal_cord_3w	SCI
SRR6655786	SCI_injured_spinal_cord_3w	SCI
SRR6655787	SCI_injured_spinal_cord_3w	SCI
SRR6655788	SCI_injured_spinal_cord_6w	SCI
SRR6655789	SCI_injured_spinal_cord_6w	SCI
SRR6655790	SCI_injured_spinal_cord_6w	SCI
SRR6655791	SCI_injured_spinal_cord_6w	SCI
SRR6655792	SCI_injured_spinal_cord_6w	SCI
SRR6655793	SCI_injured_spinal_cord_12w	SCI
SRR6655794	SCI_injured_spinal_cord_12w	SCI
SRR6655795	SCI_injured_spinal_cord_12w	SCI
SRR6655796	SCI_injured_spinal_cord_12w	SCI
SRR6655797	SCI_injured_spinal_cord_12w	SCI
SRR6655798	SCI_sham_spinal_cord_1w	CONTROL
SRR6655799	SCI_sham_spinal_cord_1w	CONTROL
SRR6655800	SCI_sham_spinal_cord_1w	CONTROL
SRR6655801	SCI_sham_spinal_cord_1w	CONTROL
SRR6655802	SCI_sham_spinal_cord_1w	CONTROL
SRR6655803	SCI_sham_spinal_cord_12w	CONTROL
SRR6655804	SCI_sham_spinal_cord_12w	CONTROL
SRR6655805	SCI_sham_spinal_cord_12w	CONTROL
SRR6655806	SCI_sham_spinal_cord_12w	CONTROL
SRR6655807	SCI_sham_spinal_cord_12w	CONTROL
SRR6789060	ctrl_spinal_cord_0d	CONTROL
SRR6789061	ctrl_spinal_cord_0d	CONTROL
SRR6789062	ctrl_spinal_cord_0d	CONTROL
SRR7232993	SCI_cont_spinal_cord_1wk	SCI
SRR7232994	SCI_cont_spinal_cord_1wk	SCI
SRR7232995	SCI_cont_spinal_cord_1wk	SCI
SRR7232996	SCI_cont_spinal_cord_1wk	SCI
SRR7232997	SCI_cont_spinal_cord_1wk	SCI
SRR7232998	SCI_cont_spinal_cord_1wk	SCI
SRR7232999	SCI_cont_spinal_cord_1wk	SCI
SRR7233000	SCI_cont_spinal_cord_1wk	SCI
SRR7233001	SCI_cont_spinal_cord_1wk	SCI
SRR7233002	SCI_cont_spinal_cord_1wk	SCI
SRR7233003	sham_spinal_cord_1wk	CONTROL
SRR7233004	sham_spinal_cord_1wk	CONTROL
SRR7233005	sham_spinal_cord_1wk	CONTROL
SRR7233006	sham_spinal_cord_1wk	CONTROL
SRR7233007	sham_spinal_cord_1wk	CONTROL
SRR789190	sham_spinal_cord_0d	CONTROL
SRR789191	sham_spinal_cord_0d	CONTROL

SRR789193	sci_contusion_spinal_cord_2d	SCI
SRR789194	sci_contusion_spinal_cord_2d	SCI
SRR789195	sci_contusion_spinal_cord_2d	SCI
SRR789196	sci_contusion_spinal_cord_1wk	SCI
SRR789198	sci_contusion_spinal_cord_1wk	SCI
SRR8327787	sham_spinal_cord_4wk	CONTROL
SRR8327788	sham_spinal_cord_4wk	CONTROL
SRR8327791	sham_spinal_cord_4wk	CONTROL
SRR8327792	sham_spinal_cord_4wk	CONTROL
SRR8327795	SCI_transection_spinal_cord_4wk	SCI
SRR8327796	SCI_transection_spinal_cord_4wk	SCI
SRR8327797	SCI_transection_spinal_cord_4wk_footprint	SCI
SRR8327798	SCI_transection_spinal_cord_4wk_footprint	SCI
SRR8327799	SCI_transection_spinal_cord_4wk	SCI
SRR8327800	SCI_transection_spinal_cord_4wk	SCI
SRR8327801	SCI_transection_spinal_cord_4wk_footprint	SCI
SRR8327802	SCI_transection_spinal_cord_4wk_footprint	SCI
SRR922121	sci_contusion_spinal_cord_1wk	SCI
SRR9332328	sham_spinal_cord_3days	CONTROL
SRR9332329	sham_spinal_cord_3days	CONTROL
SRR9332330	sham_spinal_cord_3days	CONTROL
SRR9332331	sham_spinal_cord_3days	CONTROL
SRR9332332	SCI_spinal_cord_3days	SCI
SRR9332333	SCI_spinal_cord_3days	SCI
SRR9332334	SCI_spinal_cord_3days	SCI
SRR9648454	SCI_control	CONTROL
SRR9648455	SCI_control	CONTROL
SRR9648457	SCI_control	CONTROL
