# Supplementary material for: Construction of a Searchable Database for Gene Expression Changes in Spinal Cord Injury Experiments
Source: J Neurotrauma. 2024 May 25;41(9-10):1030–43. doi: 10.1089/neu.2023.0035 (PMC11302316; doi:10.1089/neu.2023.0035)
Supplement: Supplementary Table S10 [file neu.2023.0035_suppl_tables10.pdf]

**Supplemental Table S10**: Up-regulated spinal cord genes across both mouse and rat studies, ranked by adjusted p-value. P-values and adjusted p-values not shown since they are effectively 0.

| RANK | HOMOLOGENE ID | GENE SYMBOL | GENE DESCRIPTION | CONTROL MEAN | SCI MEAN | log2FC |
|---|---|---|---|---|---|---|
| 1 | 124458 | SIGLEC1 | SIGLEC1 | 66.37 | 1477.09 | 4.476 |
| 2 | 1880 | GPNMB | GPNMB | 1466.13 | 39880.72 | 4.7656 |
| 3 | 11216 | MPEG1 | MPEG1 | 1170.47 | 12422.25 | 3.4077 |
| 4 | 2241 | CCL13 | CCL13 | 35.11 | 1706.85 | 5.6029 |
| 5 | 955 | CD68 | CD68 | 227.83 | 3919.6 | 4.1046 |
| 6 | 55936 | CD5L | CD5L | 4.79 | 184.57 | 5.2679 |
| 7 | 37832 | CST7 | CST7 | 10.15 | 458.85 | 5.4979 |
| 8 | 55616 | CTSD | CTSD | 8147.51 | 53252.95 | 2.7084 |
| 9 | 2992 | C3AR1 | C3AR1 | 81.78 | 1027.12 | 3.6505 |
| 10 | 49606 | CLEC7A | CLEC7A | 142.05 | 2641.75 | 4.217 |
| 11 | 48249 | CD84 | CD84 | 165.66 | 1941.48 | 3.5508 |
| 12 | 56783 | FAM46C | FAM46C | 134.85 | 1192.34 | 3.1443 |
| 13 | 20092 | ITGB2 | ITGB2 | 341.96 | 2243.38 | 2.7137 |
| 14 | 31315 | MAFB | MAFB | 218.06 | 1620.84 | 2.8938 |
| 15 | 11325 | GNGT2 | GNGT2 | 43.03 | 416.2 | 3.2737 |
| 16 | 37550 | CTSB | CTSB | 13175.74 | 57699.27 | 2.1306 |
| 17 | 2000 | PLEK | PLEK | 348.52 | 2946.92 | 3.0798 |
| 18 | 4067 | LGALS3BP | LGALS3BP | 556.97 | 2867.64 | 2.3641 |
| 19 | 20739 | CXCR4 | CXCR4 | 121.45 | 618.87 | 2.3492 |
| 20 | 51396 | CD300LF | CD300LF | 58.79 | 437.89 | 2.8968 |
| 21 | 30964 | NCF1 | NCF1 | 339.42 | 1767.22 | 2.3803 |
| 22 | 48514 | CD300A | CD300A | 57.75 | 528 | 3.1924 |
| 23 | 10922 | MS4A7 | MS4A7 | 15.36 | 283.27 | 4.2047 |
| 24 | 2675 | GPR65 | GPR65 | 22.56 | 229.65 | 3.3472 |
| 25 | 1022 | CTSZ | CTSZ | 925.57 | 5613.26 | 2.6004 |
