# Supplementary material for: Construction of a Searchable Database for Gene Expression Changes in Spinal Cord Injury Experiments
Source: J Neurotrauma. 2024 May 25;41(9-10):1030–43. doi: 10.1089/neu.2023.0035 (PMC11302316; doi:10.1089/neu.2023.0035)
Supplement: Supplementary Table S11 [file neu.2023.0035_suppl_tables11.pdf]

Supplemental Table S11: Down-regulated spinal cord genes across both mouse and rat studies, ranked by adjusted p-value. P-values and adjusted p-values not shown since they are effectively 0.

RANK	HOMOLOGENE ID	GENE SYMBOL	GENE DESCRIPTION	CONTROL MEAN	SCI MEAN	log2FC
1	1609	HMGCS1	HMGCS1	15519.8	4506.58	-1.784
2	30994	HMGCR	HMGCR	2372.17	1073.93	-1.1432
3	133932	MSMO1	MSMO1	3862.99	1572.33	-1.2968
4	3315	IDI1	IDI1	2586.85	658.62	-1.9736
5	55833	PPP2R2B	PPP2R2B	2748.36	1112.41	-1.3048
6	40728	HSD17B7	HSD17B7	637.14	311.71	-1.0313
7	55683	MAPK6	MAPK6	3081.64	1710.66	-0.8491
8	121937	HIGD1A	HIGD1A	1256.72	648.11	-0.9553
9	2198	RIT2	RIT2	2204.32	897.75	-1.2959
10	2355	SQLE	SQLE	2429.4	1200.26	-1.0172
11	9866	OCIAD1	OCIAD1	4397.66	2706.64	-0.7002
12	116010	YPEL3	YPEL3	3276.06	1328.61	-1.302
13	48276	ARL3	ARL3	1344.98	775.89	-0.7936
14	90913	RABL2A	RABL2A	449.69	220.77	-1.0263
15	2920	WASF1	WASF1	1509.66	585.54	-1.3663
16	5951	NSDHL	NSDHL	899.97	450.79	-0.9974
17	8822	TOX	TOX	284.75	120.15	-1.2448
18	2100	PSMD1	PSMD1	4385.72	2335.54	-0.909
19	3136	RAB9B	RAB9B	1801.46	670.97	-1.4248
20	4047	INSIG1	INSIG1	2121.42	1139.11	-0.8971
21	115930	ATXN7L3B	ATXN7L3B	4955.1	2423.57	-1.0317
22	9053	ARHGEF9	ARHGEF9	3169.59	1587.04	-0.9979
23	55488	CYP51A1	CYP51A1	5616.33	2348.79	-1.2577
24	74446	SEPT5	SEPT5	2650.75	842.83	-1.653
25	69293	AGPAT4	AGPAT4	2637.85	1235.88	-1.0938
