# Supplementary material for: Construction of a Searchable Database for Gene Expression Changes in Spinal Cord Injury Experiments
Source: J Neurotrauma. 2024 May 25;41(9-10):1030–43. doi: 10.1089/neu.2023.0035 (PMC11302316; doi:10.1089/neu.2023.0035)
Supplement: Supplementary Table S12 [file neu.2023.0035_suppl_tables12.pdf]

Supplemental Table S12: Up-regulated spinal cord genes for the mouse studies, ranked by adjusted p-value. P-values and adjusted p-values not shown since they are effectively 0.

RANK	GENE ID	GENE SYMBOL	GENE DESCRIPTION	CONTROL MEAN	SCI MEAN	log2FC
1	ENSMUSG00000029816	Gpnmb	glycoprotein (transmembrane) nmb	373.75	43221.81	6.8535
2	ENSMUSG00000035385	Ccl2	chemokine (C-C motif) ligand 2	10.89	5202.82	8.8988
3	ENSMUSG00000046718	Bst2	bone marrow stromal cell antigen 2	86.89	1933.07	4.4754
4	ENSMUSG00000069516	Lyz2	lysozyme 2	1433.11	62373.36	5.4437
5	ENSMUSG00000023992	Trem2	triggering receptor expressed on myeloid cells 2	267.13	5579.88	4.3845
6	ENSMUSG00000019987	Arg1	arginase, liver	8.28	1035.68	6.9664
7	ENSMUSG00000026728	Vim	vimentin	2013.66	16034.46	2.9932
8	ENSMUSG00000027322	Siglec1	sialic acid binding Ig-like lectin 1, sialoadhesin	47.71	1570.84	5.0409
9	ENSMUSG00000058715	Fcer1g	Fc receptor, IgE, high affinity I, gamma polypeptide	198.94	4878.42	4.6159
10	ENSMUSG00000002111	Spi1	spleen focus forming virus (SFFV) proviral integration oncogene	79.99	626.45	2.9691
11	ENSMUSG00000001403	Ube2c	ubiquitin-conjugating enzyme E2C	12.57	171.4	3.7688
12	ENSMUSG00000005413	Hmox1	heme oxygenase 1	156.73	4070.87	4.6989
13	ENSMUSG00000043157	Arl11	ADP-ribosylation factor-like 11	17.68	293.3	4.0521
14	ENSMUSG00000004707	Ly9	lymphocyte antigen 9	32.35	931.85	4.8482
15	ENSMUSG00000018774	Cd68	CD68 antigen	263.2	4256.84	4.0155
16	ENSMUSG00000024672	Ms4a7	membrane-spanning 4-domains, subfamily A, member 7	27.27	1027.41	5.2354
17	ENSMUSG00000007891	Ctsd	cathepsin D	4994.98	69743.6	3.8035
18	ENSMUSG00000040552	C3ar1	complement component 3a receptor 1	120.72	3820.48	4.984
19	ENSMUSG00000024338	Psmb8	proteasome (prosome, macropain) subunit, beta type 8 (large multifunctional peptidase 7)	76.1	742.43	3.2862
20	ENSMUSG00000030144	Clec4d	C-type lectin domain family 4, member d	6.87	470.25	6.0967
21	ENSMUSG00000030798	Cd37	CD37 antigen	94.82	639.1	2.7526
22	ENSMUSG00000041736	Tspo	translocator protein	116.01	652.14	2.4908
23	ENSMUSG00000048779	P2ry6	pyrimidinergic receptor P2Y, G-protein coupled, 6	51.19	510.52	3.3179
24	ENSMUSG00000025492	Ifitm3	interferon induced transmembrane protein 3	287.23	1539.41	2.422
25	ENSMUSG00000015950	Ncf1	neutrophil cytosolic factor 1	146.56	1727.6	3.5591
