# Supplementary material for: Construction of a Searchable Database for Gene Expression Changes in Spinal Cord Injury Experiments
Source: J Neurotrauma. 2024 May 25;41(9-10):1030–43. doi: 10.1089/neu.2023.0035 (PMC11302316; doi:10.1089/neu.2023.0035)
Supplement: Supplementary Table S13 [file neu.2023.0035_suppl_tables13.pdf]

Supplemental Table S13: Down-regulated spinal cord genes for the mouse studies, ranked by adjusted p-value. P-values and adjusted p-values not shown since they are effectively 0.

RANK	GENE ID	GENE SYMBOL	GENE DESCRIPTION	CONTROL MEAN	SCI MEAN	log2FC
1	ENSMUSG00000015401	Cltrn	collectrin, amino acid transport regulator	26.32	1.25	-4.387
2	ENSMUSG00000028307	Aldob	aldolase B, fructose-bisphosphate	166.24	10.71	-3.9558
3	ENSMUSG00000041992	Rapgef5	Rap guanine nucleotide exchange factor (GEF) 5	1270.47	716.43	-0.8264
4	ENSMUSG00000023914	Mep1a	meprin 1 alpha	66.79	1.27	-5.7056
5	ENSMUSG00000061742	Slc22a12	solute carrier family 22 (organic anion/cation transporter), member 12	33.98	1.98	-4.0959
6	ENSMUSG00000026721	Rabgap1l	RAB GTPase activating protein 1-like	1386.8	782.41	-0.8257
7	ENSMUSG00000033769	Exoc6b	exocyst complex component 6B	1605.07	1132.36	-0.5033
8	ENSMUSG00000093930	Hmgcs1	3-hydroxy-3-methylglutaryl-Coenzyme A synthase 1	5196.84	2551.5	-1.0262
9	ENSMUSG00000049764	Zfp280b	zinc finger protein 280B	239.76	167.53	-0.5171
10	ENSMUSG00000012076	Brms1l	breast cancer metastasis-suppressor 1-like	805.73	494.99	-0.7028
11	ENSMUSG00000024378	Stard4	StAR-related lipid transfer (START) domain containing 4	611.57	318.82	-0.9397
12	ENSMUSG00000063903	Klk1	kallikrein 1	18.84	0.34	-5.7692
13	ENSMUSG00000031029	Eif3f	eukaryotic translation initiation factor 3, subunit F	12759.02	2274.54	-2.4878
14	ENSMUSG00000027359	Slc27a2	solute carrier family 27 (fatty acid transporter), member 2	373.44	90.71	-2.0414
15	ENSMUSG00000029221	Slc30a9	solute carrier family 30 (zinc transporter), member 9	2325.52	1729.25	-0.4274
16	ENSMUSG00000049106	Dcaf5	DDB1 and CUL4 associated factor 5	884.99	686.48	-0.3664
17	ENSMUSG00000109982	Gm45520	predicted gene 45520	4.57	1.85	-1.3003
18	ENSMUSG00000046157	Tmem229b	transmembrane protein 229B	874.47	566.45	-0.6264
19	ENSMUSG00000028926	Cdk14	cyclin-dependent kinase 14	2232.34	1287.2	-0.7943
20	ENSMUSG00000045294	Insig1	insulin induced gene 1	1469.38	764.67	-0.9423
21	ENSMUSG00000052539	Magi3	membrane associated guanylate kinase, WW and PDZ domain containing 3	766.42	465.29	-0.7199
22	ENSMUSG00000039262	Prrc2b	proline-rich coiled-coil 2B	4431.63	2719.75	-0.7043
23	ENSMUSG00000021775	Nr1d2	nuclear receptor subfamily 1, group D, member 2	1530.45	983.72	-0.6376
24	ENSMUSG00000062794	Zfp599	zinc finger protein 599	44.41	24.55	-0.8551
25	ENSMUSG00000054843	Atrnl1	attractin like 1	1040.71	551.55	-0.916
