# Supplementary material for: Construction of a Searchable Database for Gene Expression Changes in Spinal Cord Injury Experiments
Source: J Neurotrauma. 2024 May 25;41(9-10):1030–43. doi: 10.1089/neu.2023.0035 (PMC11302316; doi:10.1089/neu.2023.0035)
Supplement: Supplementary Table S14 [file neu.2023.0035_suppl_tables14.pdf]

Supplemental Table S14: Up-regulated spinal cord genes for the rat studies, ranked by adjusted p-value. P-values and adjusted p-values not shown since they are effectively 0.

RANK	GENE ID	GENE SYMBOL	GENE DESCRIPTION	CONTROL MEAN	SCI MEAN	log2FC
1	ENSRNOG00000021243	Siglec1	sialic acid binding Ig like lectin 1	61.47	1437.01	4.5468
2	ENSRNOG00000046683	Lilrb3	leukocyte immunoglobulin like receptor B3	33.6	454.67	3.7581
3	ENSRNOG00000008816	Gpnmb	glycoprotein nmb	2292.52	37277.49	4.0232
4	ENSRNOG00000054251	Clec7a	C-type lectin domain containing 7A	128.06	2767.95	4.4338
5	ENSRNOG00000030187	Mmp12	matrix metallopeptidase 12	9.61	743.74	6.2736
6	ENSRNOG00000037563	Cd68	Cd68 molecule	192.33	3609.94	4.2302
7	ENSRNOG00000006583	Hpgds	hematopoietic prostaglandin D synthase	13.7	163.38	3.5753
8	ENSRNOG00000016037	Mafb	MAF bZIP transcription factor B	123.36	715.13	2.5352
9	ENSRNOG00000047367	Card14	caspase recruitment domain family, member 14	75.45	905.56	3.5851
10	ENSRNOG00000001959	Mx1	myxovirus (influenza virus) resistance 1	178.35	1266.11	2.8275
11	ENSRNOG00000020805	Sost	sclerostin	2.21	85.24	5.2654
12	ENSRNOG00000007129	Cd8b	CD8b molecule	6.76	147.49	4.4465
13	ENSRNOG00000021084	AABR07006310.1	macrophage expressed 1	1153.8	7274.69	2.6564
14	ENSRNOG00000021242	Adam33	ADAM metallopeptidase domain 33	13.69	158.03	3.5288
15	ENSRNOG00000008045	Slamf9	SLAM family member 9	24.47	251.65	3.3619
16	ENSRNOG00000049282	Oas2	2'-5' oligoadenylate synthetase 2	14.11	80.39	2.5099
17	ENSRNOG00000006108	Gngt2	G protein subunit gamma transducin 2	35.72	290.98	3.026
18	ENSRNOG00000045558	Cd34	CD34 molecule	268.57	970.79	1.8538
19	ENSRNOG00000018126	Abca1	ATP binding cassette subfamily A member 1	2404.49	12129.48	2.3347
20	ENSRNOG00000027811	Lilrb4	leukocyte immunoglobulin like receptor B4	83.33	2104.98	4.6587
21	ENSRNOG00000026607	Tnfsf18	TNF superfamily member 18	5.16	85.7	4.0519
22	ENSRNOG00000015024	Mcoln3	mucolipin 3	15.79	118.82	2.9115
23	ENSRNOG00000017625	Htr2b	5-hydroxytryptamine receptor 2B	22.19	293.66	3.7256
24	ENSRNOG00000008933	Plbd1	phospholipase B domain containing 1	82.96	737.56	3.1521
25	ENSRNOG00000018426	NEWGENE_2134	apolipoprotein C1	29.78	211.37	2.8271
