# Supplementary material for: Construction of a Searchable Database for Gene Expression Changes in Spinal Cord Injury Experiments
Source: J Neurotrauma. 2024 May 25;41(9-10):1030–43. doi: 10.1089/neu.2023.0035 (PMC11302316; doi:10.1089/neu.2023.0035)
Supplement: Supplementary Table S15 [file neu.2023.0035_suppl_tables15.pdf]

Supplemental Table S15: Down-regulated spinal cord genes for the rat studies, ranked by adjusted p-value. P-values and adjusted p-values not shown since they are effectively 0.

RANK	GENE ID	GENE SYMBOL	GENE DESCRIPTION	CONTROL MEAN	SCI MEAN	log2FC
1	ENSRNOG00000061507	AABR07032328.1	AABR07032328.1	112.7	1.21	-6.5382
2	ENSRNOG00000031098	AABR07022157.1	AABR07022157.1	50.92	1.23	-5.3632
3	ENSRNOG00000007206	LOC361016	similar to RIKEN cDNA 4933406L09	3281.56	1103.46	-1.5723
4	ENSRNOG00000023182	AABR07030085.1	AABR07030085.1	42.78	1.53	-4.798
5	ENSRNOG00000017167	AABR07033851.1	AABR07033851.1	46.39	0.9	-5.6763
6	ENSRNOG00000016122	Hmgcr	3-hydroxy-3-methylglutaryl-CoA reductase	3382.7	1335.81	-1.3404
7	ENSRNOG00000048750	AABR07047576.1	AABR07047576.1	27.76	1.3	-4.4162
8	ENSRNOG00000015428	Mff	mitochondrial fission factor	4640.43	755.47	-2.6187
9	ENSRNOG00000014105	AC103056.1	AC103056.1	22.68	0.31	-6.1764
10	ENSRNOG00000059756	AABR07029023.1	AABR07029023.1	37.17	0.71	-5.6967
11	ENSRNOG00000056748	AC229945.1	AC229945.1	25.86	0.82	-4.9689
12	ENSRNOG00000032297	Msmo1	methylsterol monooxygenase 1	6058.78	2384.03	-1.3456
13	ENSRNOG00000051341	AABR07038849.1	AABR07038849.1	549.66	178.08	-1.6259
14	ENSRNOG00000029083	AC106932.1	AC106932.1	50.53	0.34	-7.2038
15	ENSRNOG00000031401	AC107280.1	AC107280.1	32.4	0.65	-5.6295
16	ENSRNOG00000057738	AC104053.1	AC104053.1	30.39	0.5	-5.905
17	ENSRNOG00000006989	Vamp2	vesicle-associated membrane protein 2	11514.41	2294.81	-2.3269
18	ENSRNOG00000012345	AABR07004276.1	similar to 60S ribosomal protein L26	25.11	1.19	-4.3945
19	ENSRNOG00000062305	AABR07013388.1	AABR07013388.1	23.66	0.46	-5.6606
20	ENSRNOG00000007658	AC103101.1	AC103101.1	55.79	0.15	-8.474
21	ENSRNOG00000030738	LOC100362040	Ac2-143-like	21.39	1.09	-4.2948
22	ENSRNOG00000059854	AABR07012139.1	AABR07012139.1	30.62	1.91	-3.9968
23	ENSRNOG00000016552	Hmgcs1	3-hydroxy-3-methylglutaryl-CoA synthase 1	24311.75	6717.55	-1.8556
24	ENSRNOG00000002826	Hsd17b7	hydroxysteroid (17-beta) dehydrogenase 7	745.02	277.35	-1.4255
25	ENSRNOG00000047992	AABR07013410.1	AABR07013410.1	31.6	0.3	-6.7148
